# Empirical modelling of 2205 DSS flow curves using strain-compensated Arrhenius rate-type constitutive model

**DOI:** 10.1038/s41598-024-72441-9

**Published:** 2024-09-28

**Authors:** Elvis M. Gonya, Charles W. Siyasiya, Mamookho E. Makhatha

**Affiliations:** 1 Department of Metallurgy, Faculty of Engineering and the Built Environment, University of Johannesburg, John Orr Building, Doornfontein, Johannesburg, 2028 South Africa; 2 Department of Materials Science and Metallurgical Engineering, University of Pretoria, Hatfield, Pretoria, 0028 South Africa

**Keywords:** 2205 duplex stainless steel, Gleeble, Hot deformation constants, Generalised reduced gradient, Predictive model, Non-linear model, Engineering, Materials science, Mathematics and computing

## Abstract

This work predicts, hot flow curves of 2205 DSS using strain-compensated Arrhenius rate-type constitutive model. Twenty-five (25) × **Ø**10 diameter × 15 mm height cylindrical samples were hot compressed at a temperature between 850 and 1050 °C at an interval of 50 °C and strain rates between 0.001 and 5 s^−1^, using Gleeble 1500D. After the tests, corrected flow curves were plotted followed by computation of deformations constants at various deformation conditions using steady state stress. The values of the constants were (α = 0.009708, Q = 445 kJ/mol and n = 3.7) and seemed comparable to the previous studies of DSS. Steady state predictive model was then constructed using the calculated constants and showed a reasonably good accuracy with low value of MARE = 7.78%. Furthermore, calculated strain compensated Arrhenius rate type model was used to predict flow curves at various deformation. The model had a good estimation of flow curves of flow curves at 900–1050 °C across all strain rates as reflected by MARE = 5.47%. A notable discrepancy between predicted and experimental flow stress was observed at 850 °C and across all the strain rates. A model refinement using generalised reduced gradient improved the accuracy of the model by 34.7% despite deformation conditions at 850 °C and low strain rates (0.01/ 0.1) s^−1^ showing minimum improvement. Further modification of Z-parameter by compensating for the strain rate improved the accuracy of the model at 850 °C/0.01 s^−1^/0.1 s^−1^. Lastly, a comparison of the current model with the other non-linear model showed that the latter was more accurate in estimation of flow curves since it relied on characteristics flow stress points controlled by underlying active deformation mechanisms.

## Introduction

The 2205 duplex stainless steel (DSS) is a dual phase alloy with approximately equal proportion of austenite and delta-ferrite phase^1^. The existence of dual phase in this alloy is ascribed to the manner in which major alloying elements (chromium, nickel, and molybdenum) partitioned between austenite and ferrite phases^2^. The dual phase structure gives the alloy good intrinsic and functional mechanical properties compared to single phase austenite or ferritic stainless steels^3,4^. As such, the 2205 DSS finds its popularity in several engineering applications including marine, sugar-mills, petroleum, oil and gas industries, construction, and paper mills^5–7^.

The good mechanical properties (e.g., yield strength, toughness, corrosion resistance) embedded in 2205 DSS are mostly achieved during thermo-mechanical processing (TMP)^1,8^. TMP is a thermal treatment that uses both heat and mechanical forces under varied deformation conditions to refine the deformed microstructure^9–11^. During this process, it is thought that dynamic restoration mechanisms such as dynamic recrystallisation (DRX) and dynamic recovery (DRV) are activated^12^. And it is these mechanisms that are responsible for enhancing the mechanical properties of 2205 DSS through microstructural refinement^11,13,14^. The 2205 DSS contains two phases with different rheological behavior, as such it has been well documented that these phases experience different restoration mechanisms during hot rolling^15–17^. Given that the deformation of DSS occur in the two-phase region, the DRV and DRX are likely to occur ferritic and austenitic phase respectively^18^. Occurrence of DRV or DRX occur in each phase, is controlled by the level of stacking fault energies (SFE)^19^. In austenitic phase, the SFE level are deemed to be low and thus thermal activated slip process such as cross slip and climbing of dislocations are restricted^18^. In this sense, dislocations generated during plastic deformation are able to build up to a critical level where restoration of austenite phase by DRX becomes feasible^14^. On the other hand, ferritic phase has high SFE and of which this allows the thermal activated slip process to be easier and subsequently the phase recover via the formation of subgrains inside the deformed ferrite grain. Despite the type of restoration mechanism taking place in each phase, the final hot rolled product has a refined microstructure with improved mechanical properties.

Since the restoration mechanisms are of practical importance when hot processing the alloys, it is crucial to establish metallurgical tools that are able to predict whether these mechanisms are initiated during hot working. To this end, the most useful tool is the hot flow curve generated during plastic deformation. Flow curves give an indication of how a deforming alloy respond to imposed deformation parameters. In other words, they give a clue on the type of restoration/deformation mechanisms governing the microstructural development of the alloy. Indeed, in most TMP studies^14,20–24^, flow curves are constructed and analysed to identify the types of restoration mechanisms that are at play during hot working. For instance, it is generally accepted that hot flow curves that show single or multiple peaks post work hardening followed by a drop in flow stress till steady state represent DRX behavior^11^. Whereas those that show no peak in flow stress but rather maintained a plateau in flow stress after work hardening display a DRV behaviour^20,25^.

On the basis of the importance of flow curves, significant research devoted to modelling of flow curves using constitutive models have been conducted^26–30^. In turn, the predictive models have been integrated into hot rolling programs to predict hot flow behaviour of alloys under varied deformation conditions^27^. The predicted flow curves are vital for hot metal processing industries as they do not only predict restorative mechanisms but also assist in estimation of rolling forces and process optimisation^13,31–33^. However, despite the significant work conducted on modelling of flow curves, the 2205 DSS has received less attention. And it is in this view that this paper seeks to lessen the gap and employ strain compensated constitutive model to predict the hot flow curves of 2205 DSS at various deformation conditions.

## Materials and methods

**Fig. 1:**
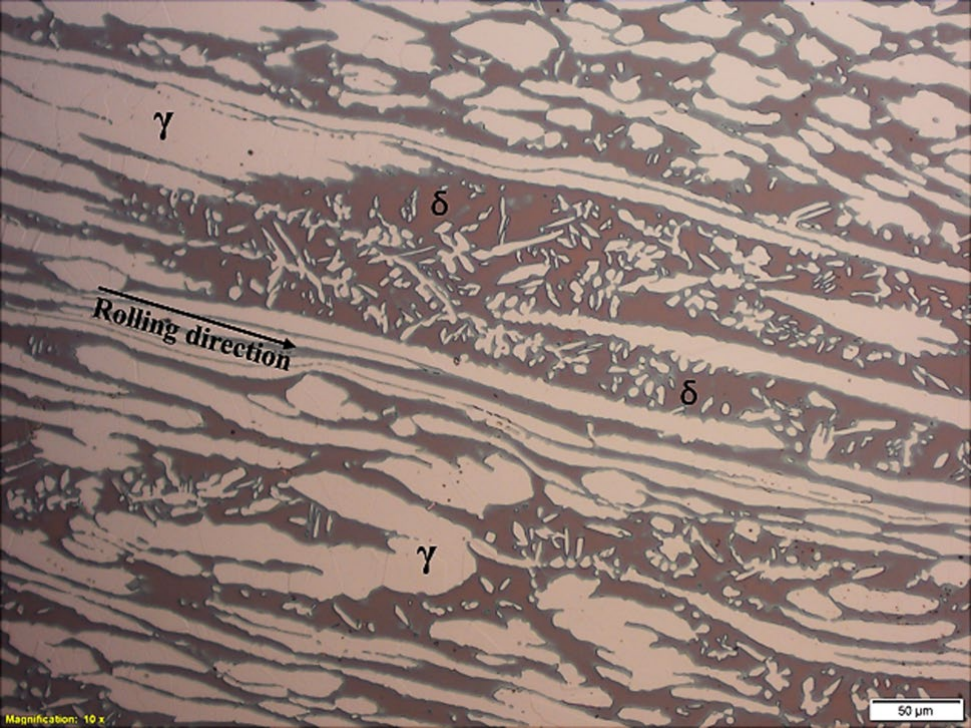
2205 DSS as-received microstructure with ferrite matrix (brown) and austenite islands (light phase).

In this study, the as-rolled 2205 DSS in the form of Ø10mm rod by 1 m was acquired from Multi Alloys (LTD). The composition of the alloy in (wt%) is given in Table 1. The as-received microstructure had a phase balance of 51% ferrite (brown matrix) and 49% austenite phase, which appear as light islands distributed along the rolling direction within the ferrite matrix as shown in Fig. 1.

**Table 1:** Composition of as-received 2205 DSS.

%C	%Si	%Mn	%P	%S	%Cr	%Ni	%Mo	%N	%Fe
0.023	0.34	1.67	0.029	0.003	22.80	5.20	3.20	0.1703	66.57

**Fig. 2:**
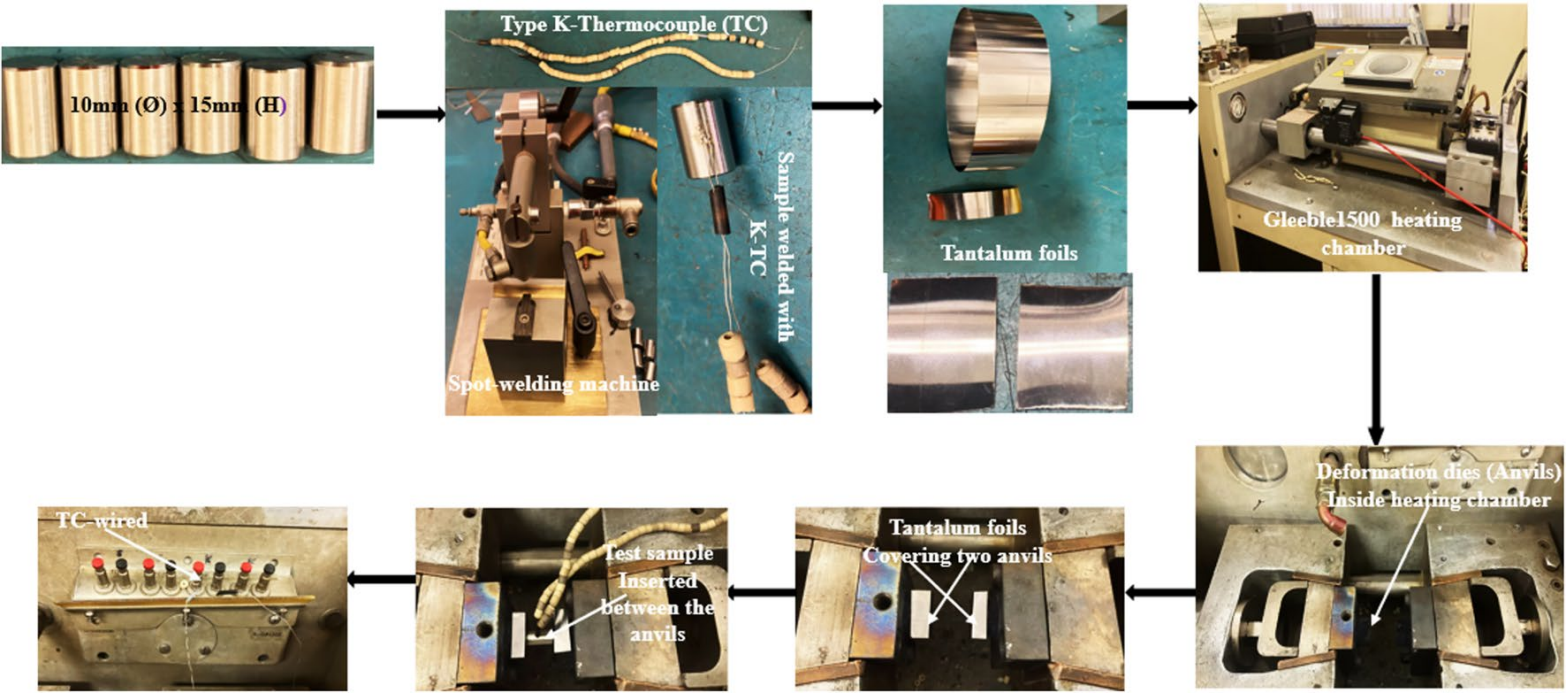
Schematic representation of sample preparation before hot compression testin a Gleeble 1500.

**Fig. 3:**
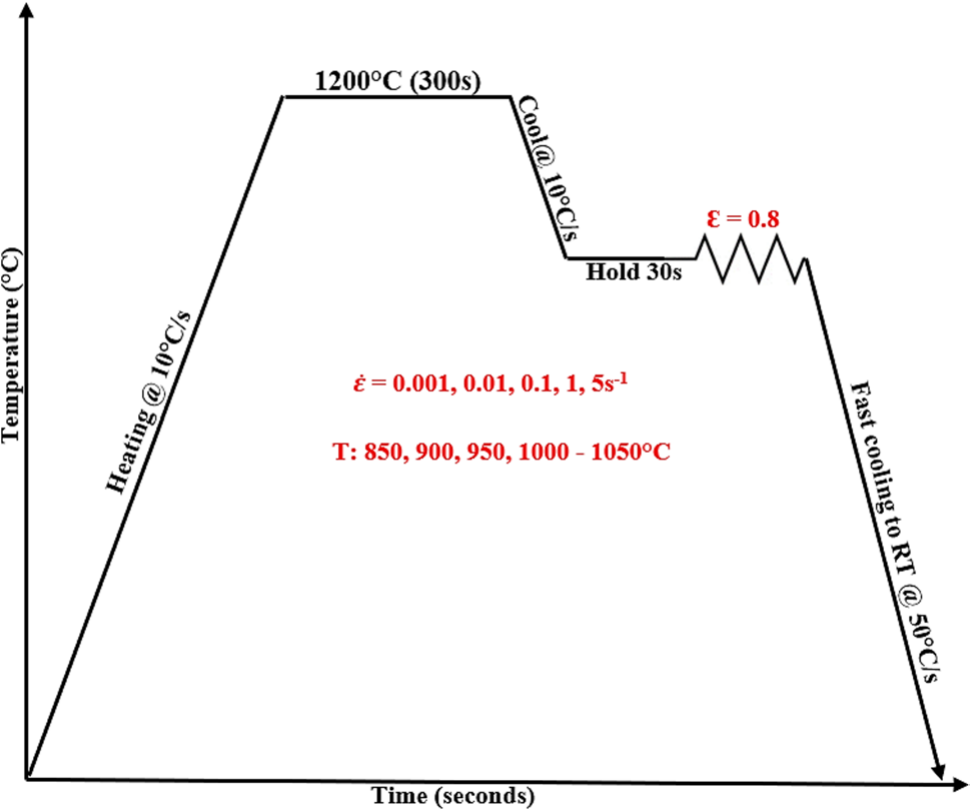
Hot compression deformation cycle of 2205 DSS.

The Ø10mm rod was sectioned into pieces of 15 mm height to make **Ø**10mm x 15 mm height cylindrical samples that meet the requirements of Gleeble 1500 thermomechanical simulator. Before conducting the single-hit hot compression tests, each sample was prepared according to Figure 2. The k-type thermocouple was spot welded at the mid-height of each sample, parallel to the loading axis to monitor the actual deformation temperature during testing. The effect of friction was managed by placing two sizeable sheets of tantalum foil between deformation dies and the specimen. Samples were then inserted into the heating chamber and k-thermocouple attached to temperature control unit. Following the deformation cycle presented in Fig. 3 below, twenty-five hot compression tests were conducted between the temperatures of 850–1050 °C at 50 °C interval, strain rate of 0.001–5 s^−1^, and to a total true strain of 0.8. Following the hot compression tests, some of the deformed samples were initially sectioned parallel to the loading axis, mounted and mechanical grounded in water using silicon abrasive paper from 240 to 1200 Grit size. They were then polished using 3 μm and 1 µm diamond paste and finalised with colloidal silica. After polishing, samples were thoroughly cleaned with water, dried with ethanol before being electro-etched for 20-25 s in a 60% HNO_3_ solution. Post metallographic preparation microstructural analysis was done on each sample using Olympus DSX-CB optical microscope.

To improve the reliability of the stress–strain data after hot compression tests, the influence of friction generated in each test was assessed by calculating the barrelling factor (B) using the procedure described by Sen Hao et al.^30^. All tests had a B value that was greater 1.1^34^, which then necessitated the adjustment of flow curves for friction as per mathematical procedure presented by Annan et al.^35^. Adiabatic heating of samples tested at higher strain rates of 1 and 5 s^−1^ was confirmed through significant temperature difference between the programmed and actual temperature measured by the thermocouple. As a result, flow curves pertaining to these strain rates were corrected for adiabatic heating using a general procedure described by several researchers^36–38^. After correcting for both friction and adiabatic heating, flow curves were plotted using Origin software 2022, followed by constitutive analysis of hot deformation constants.

## Results and discussion

### Flow curves

**Fig. 4:**
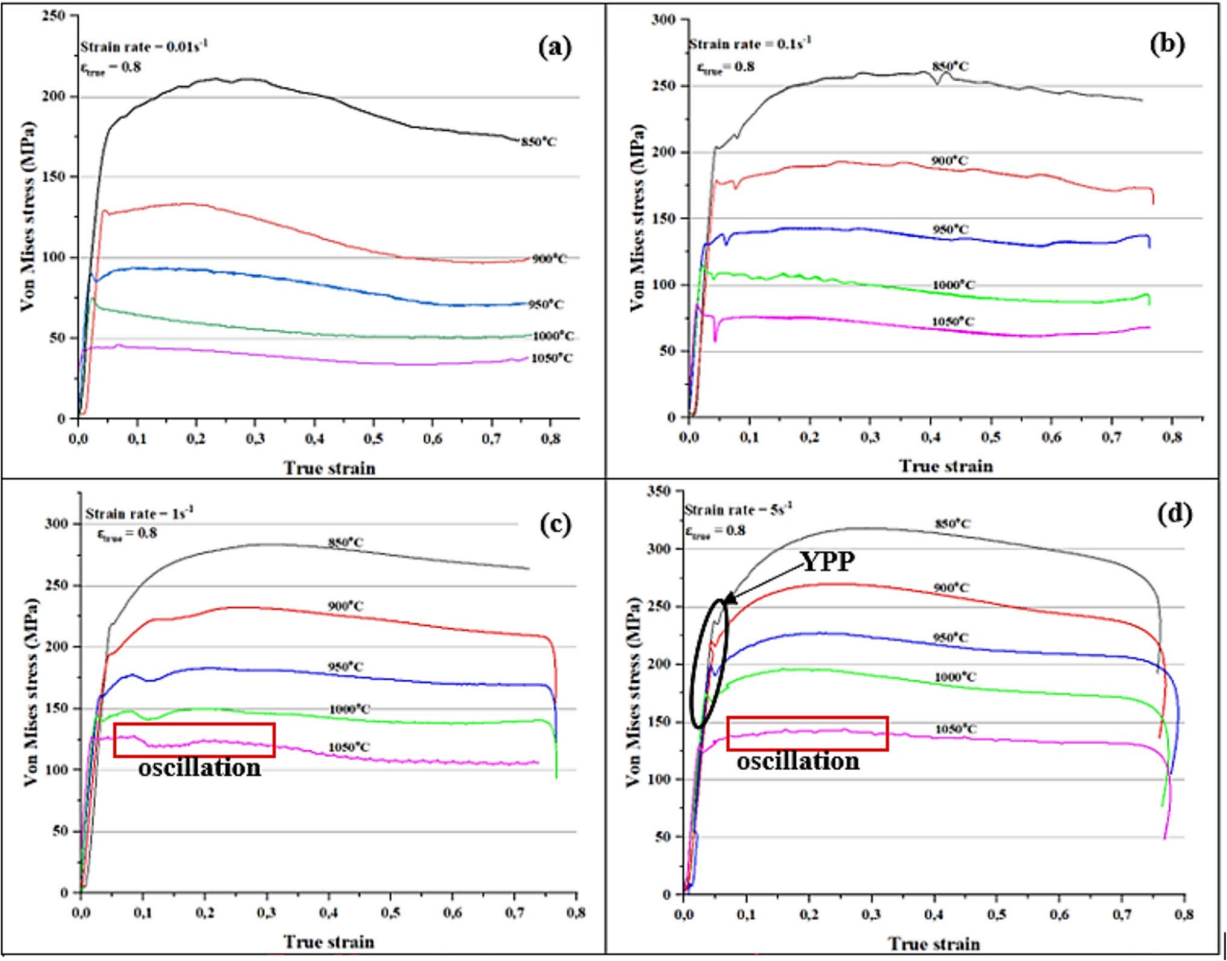
Experimental flow curves of 2205 DSS at various hot working conditions.

The 2205 DSS flow curves that were obtained from the single-hit hot compression tests are presented in Figure 4. The influence of strain rate and temperature on the material’s response is well documented in literature. Where, both deformation parameters act opposite to bring a similar response in a deforming alloy. Flow curves showed that an increase in strain rate at constant temperature or decrease in temperature at constant strain rate brought an increase in flow stress, and vice versa, During hot deformation, the material’s response to imposed deformation conditions is mainly influenced by the amount of dislocation generated during hot forming process. In the early stage of deformation as shown by the flow curves in Figure 4 below, it was observed that the alloy experienced increase in flow stress till attainment of peak point due to work hardening. The increase in flow stress could be ascribed to increase in dislocation density of the alloy, where some dislocations become immobile and act as obstacles to moving dislocations. This phenomenon increases the resistance of the alloy to slip and necessitate the increased in applied stress for continued deformation. Post peak point, it was also noticed that the alloy either maintained a plateau or a drop in flow stress till attainment of steady state. The manner in which the alloy responded from the peak could be attributed to dominant restorative mechanisms that was active at a particular deformation condition. The following discussion looked at how the changes in imposed hot processing parameters interact with softening mechanisms that govern the microstructural evolution during hot working.

**Fig. 5:**
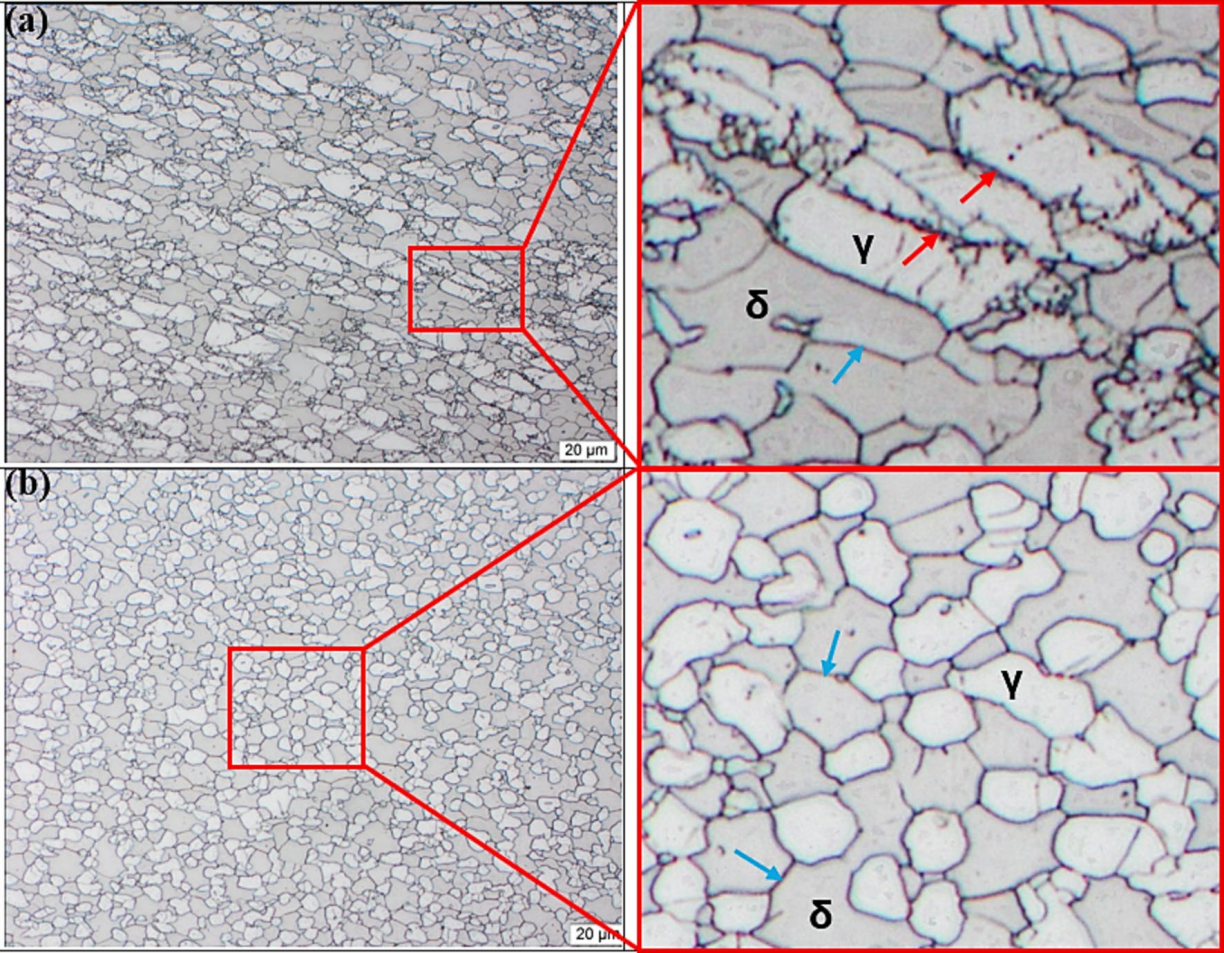
Optical micrographs of 2205 DSS at: (**a**) 900 °C and (**b**) 1000 °C for a strain rate of 0.01 s-1. and total true strain of 0.8.

At a strain rate of 0.01 s^−1^ (Fig. 4a) the drop in flow stress was observed at a true strain below 0.1 for 950–1050 °C, whereas for 850–900 °C the drop occurred at a true strain of 0.2. The observed reduction in flow stress from the peak point at respective deformation conditions could have come as a result of softening by means DRX. Higher temperatures also showed earlier drop when compared to lower temperatures possibly due to decrease in critical strain and increase in migration rate of grain boundaries from additional thermal energy. The occurrence of softening was confirmed through optical micrographs shown in Figure 5. At 900 °C/0.01 s^−1^ (see Figure 5a) the microstructure revealed elongated austenite grains (white phase) largely distributed along the ferrite matrix (brown phase). The deformation substructures (indicated by red arrows) and some signs of nucleation were also seen on the surface of austenite grains suggesting partial recrystallization took place. Moreover, the ferrite matrix also experienced some softening as indicated by well-defined high angle grain boundaries (HAGBs) (pointed by blue arrows). By increasing the temperature to 1000 °C at the same strain rate, full recrystallization of austenitic phase was realised as shown in Figure 5b. Unlike at lower temperature the austenite grains were more equiaxed with no evidence of deformation substructures or mechanical twins. It is worth noting that, despite the full recrystallization, the grain size distribution of equiaxed austenite grains was not uniform, and this was attributed to the rate of nucleation and growth of recrystallised grains at lower strain rate. Unlike at higher strain rates the nucleation process first occurs on a relatively few nuclei when the strain rates are lower, subsequently grains that form first grow in size until they start to impinge and restrict growth of newly formed grains. Hence the microstructure with inhomogeneous austenite grain size distribution was observed at 1000 °C/0.01 s^−1^. Manshadi et.al^36^, also observed a similar phenomenon where the growth rate of some recrystallised grains occurred faster and caused impingement and growth restrictions on smaller grains.

**Fig. 6:**
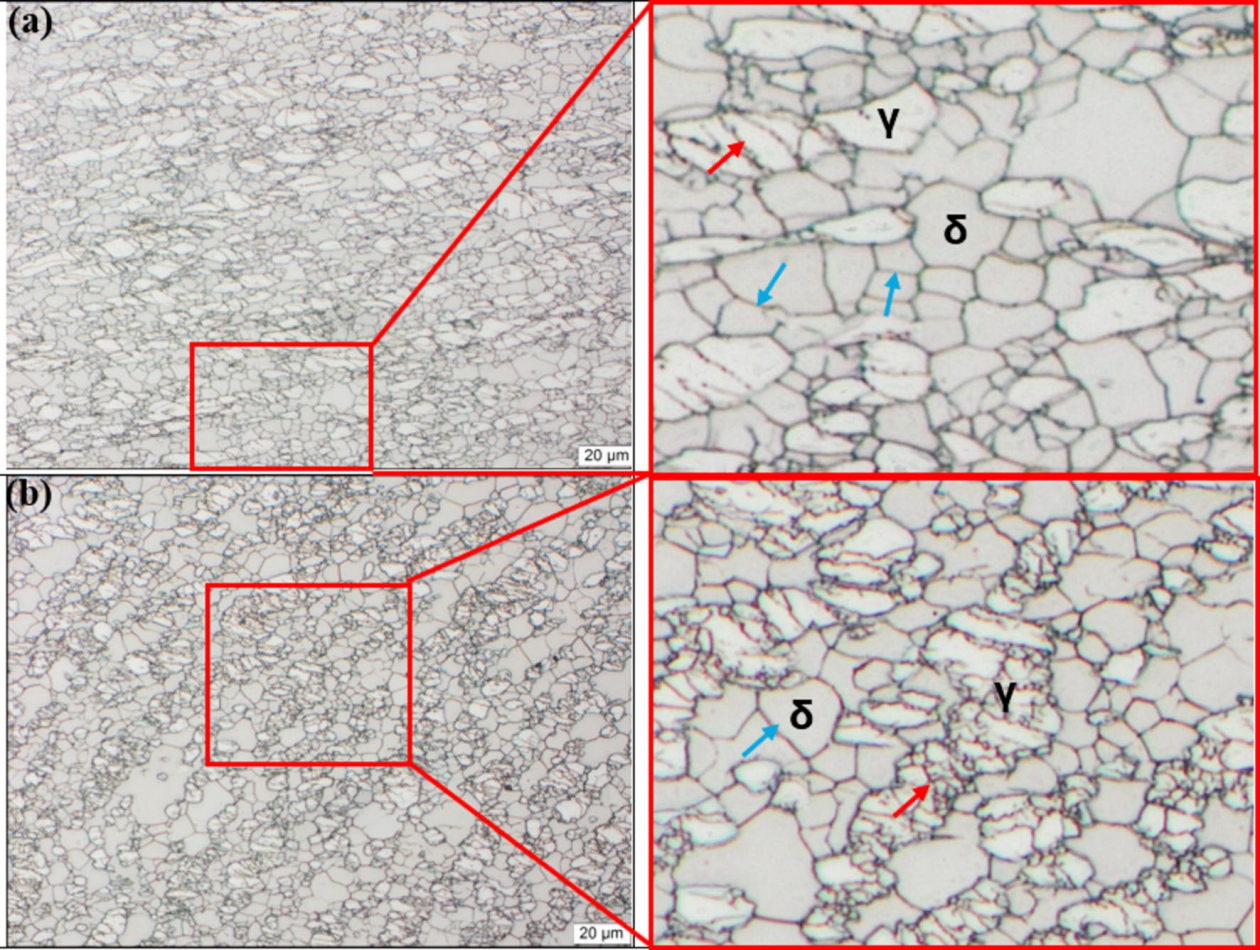
Optical micrographs of 2205 DSS at: (**a**) 900 °C and (**b**) 1000 °C for a strain rate of 5 s-1 and total true strain of 0.8.

At a strain rate of 1 s^−1^ and temperature range between 900 and 1050 °C, a peak at a strain of approximately 0.05 and again at 0.2. According to Han J et al.^37^, multiple peaks could be due to strain distribution between ferrite and austenite, where at higher strains the load transfer to hard austenite may cause the alloy to re-work hardened, resulting in the secondary peak stress. With increase in strain rate to 5 s^−1^, all the flow curves showed a broad peak followed by softening, however interpretation of the drop at higher strain rates must be done with caution since adiabatic heating also manifest itself in the form of a drop in flow stress. In this view, microstructural analysis was done to determine the cause of softening as well as to rule out the occurrence of flow instabilities. Figure 6 shows optical micrographs that were prepared at 900 °C (a) and 1000 °C (b) for a strain rate of 5 s^−1^ and it can be seen that both deformation conditions induced substantial softening of ferrite matrix. At this level of strain rate, DRV in ferrite matrix was limited due to shorter time available for subgrain formation. As a result, partial softening occurred creating a strain energy gradient in the matrix that provides the driving force for strain induced boundary migration (SIBM). This condition led to softening of ferrite matrix by means of discontinuous dynamic recrystallisation (DDRX). Similar findings related to restoration of ferrite by DDRX at higher strain rates were also reported by Chen et al.^38^, and Haghdadi et al.^39^, in duplex stainless steel studies. On the other hand, at lower temperature of 900 °C, the deformed austenite grains surfaces revealed nucleation process taking place at the deformation substructures (see red arrows). And with increase in temperature to 1000 °C, the nucleation process dominated and spread to the rest of austenite grains, suggesting that an increased in temperature may have enhanced the kinetics of recrystallization.

Another interesting observation was a yield point phenomenon (YPP) just after work-hardening in Figure 4d which is a flow curve obtained at a strain rate of 5 s^−1^. According to^40–42^, the occurrence of YPP can be attributed to several factors including: (1) emission of new dislocations from the grain boundaries, (2) increase in dislocation density as a result of unpinning of dislocations from the solutes, and (3) strain transfer from austenite to ferrite to name a few. The YPP is more prevalent at low temperatures and it tends to disappear with increase in temperature^43^. At high temperatures, atoms are able to diffuse faster and match the velocity of gliding dislocations, hence the disappearance of YPP. At a temperature of 1050 °C, (1 and 5 s^−1^) oscillation behaviour of the alloy was also noticed after work hardening. A possible explanation to this behaviour is that grains which were recrystallised earlier may have experienced the second cycle of work hardening and recrystallisation, hence the observation of oscillations from the flow curve^44^. The other flow curves (Fig. 4b,c) showed a faint peak after work hardening suggesting sluggish DRX kinetics or dominance of dynamic recovery (DRV) process, this was mainly observed at high temperatures (1000–1050 °C). The reason could be that at higher temperatures, the volume fraction of ferrite is expected to be higher due to solid phase transformation of γ to δ, hence the suspected dominance of extended DRV.

## Computation of hot deformation constants

The material’s response in terms of flow stress largely depends on the imposed deformation conditions $\left(T, \dot{\varepsilon }, and \varepsilon \right)$, where T and $\dot{\varepsilon }$ are said to said to play a major role^45^. For instance, fluctuations in flow stress are brought by the combined effect of temperature and strain rate, which happens to act in the opposite direction to bring a similar response in a deforming alloy. To this end, this combined effect of two deformation parameters (T and $\dot{\varepsilon })$ is widely represented by a single parameter known as Zener–Holloman (Z):

1 $$Z=\dot{\varepsilon }\mathit{exp}\left(\frac{Q}{RT}\right)$$

where: $\dot{\varepsilon }$ = strain rate in s^−1^, Q = deformation activation energy in kJ/mol, R = universal gas constant = 8.314 J mol^−1^ K^−1^, and T = deformation temperature in Kelvin.

### Constitutive models of hot deformation

According Sellars and Tergat^46^, steady state creep models previously developed by Garofalo can be adopted to hot working studies. In this sense, the relationship between the material’s response and the deformation conditions can be represented by means of hyperbolic sine equation:

2 $${\varvec{Z}}={{\varvec{A}}}_{1}\{{{\varvec{sinh}}(\boldsymbol{\alpha }{\varvec{\sigma}})\}}^{{\varvec{n}}}$$

where: A_1_ = microstructural parameter in s^-1^, α = stress multiplier, and n = stress exponent.

Equation (2) covers a wide range of stress, however the hot working process can be evaluated at high or low stress levels. From this, Sellars and Tergat^46^ suggested the division of the hyperbolic sine equation into two forms. Where for low and high stress region, the equation was transformed into power law and exponential form respectively. Thus, the power law is given as follows:

3 $$\dot{\varepsilon }={{A}_{2}\sigma }^{{n}{\prime}}\mathit{exp}\left(-\frac{Q}{RT}\right)$$

and the exponential law is

4 $$\dot{\varepsilon }={A}_{3}exp\left(\beta \sigma \right)\mathit{exp}\left(-\frac{Q}{RT}\right)$$

Equation (2) can be manipulated further to give a constitutive model necessary for predicting the flow stress any particular true strain in a flow curve^16,28,47^. Initially, flow stress from Eq. (2) can be expressed as:

5 $$\sigma =\frac{1}{\alpha }{\mathit{sin}h}^{-1}{\left[\frac{Z}{A}\right]}^\frac{1}{n}$$

whereby the inverse hyperbolic function of ${\text{sinh}}^{-1}x$ = $\text{ln}\left[x + \sqrt{{x}^{2}+1}\right]$, thus substituting of this function into Eq. ((5), considering ${\left[\frac{Z}{A}\right]}^\frac{1}{n}$ = x yields Eq. ((6):

6 $$\sigma_{(ssorpeakstress)} = 1/\alpha \ln \left\{ {(Z/(A_{1} ))^{(1/n)} + [(Z/(A_{1} ))^{(2/n)} + 1]^{(1/2)} } \right\}(MPa)$$

Depending on what is being modelled, Eq. ((6) can be used to predict characteristics stresses (critical, peak, or steady state) in a flow curve. However, hot deformation constants including (Q, α, ${A}_{1}$ and n) must first be calculated in order to predict the characteristic stress. Such calculations require systematic manipulation of Eqs. ((*2*), ((*3*) and ((*4*) as explained below^48^:

#### Computation of stress multiplier (α)

Α is known as stress multiplier and can be calculated using the ratio of β and n’ (α =$\frac{\beta }{{n}{\prime}}$) constants found in Eq. ((4) and Eq. ((3) respectively. By expressing natural logarithms on both sides of Eq. ((3) and Eq. ((4), the following linear logarithmic equations results:

7 $$\ln \varepsilon = n^{\prime}\ln \sigma + \underbrace {{\ln A_{2} - \frac{Q}{RT}}}_{cons\tan t}$$

where: $$n^{\prime} = (\partial (\ln \dot{\varepsilon })/\partial (\ln \sigma )\left| {_{T,\varepsilon } } \right. = gradientofthelinearcurveatcons\tan t{\text{Tan}} d\varepsilon$$.

And:

8 $$\ln \varepsilon = \beta \sigma + \underbrace {{\ln A_{3} - \frac{Q}{RT}}}_{cons\tan t}$$

where $$\beta = \frac{{\partial \left( {\ln \dot{\varepsilon }} \right)}}{\partial \sigma }\left| {_{T,\varepsilon } } \right. = gradientofthelinearcurveatcons\tan t{\text{Tan}} d\varepsilon$$

**Fig. 7:**
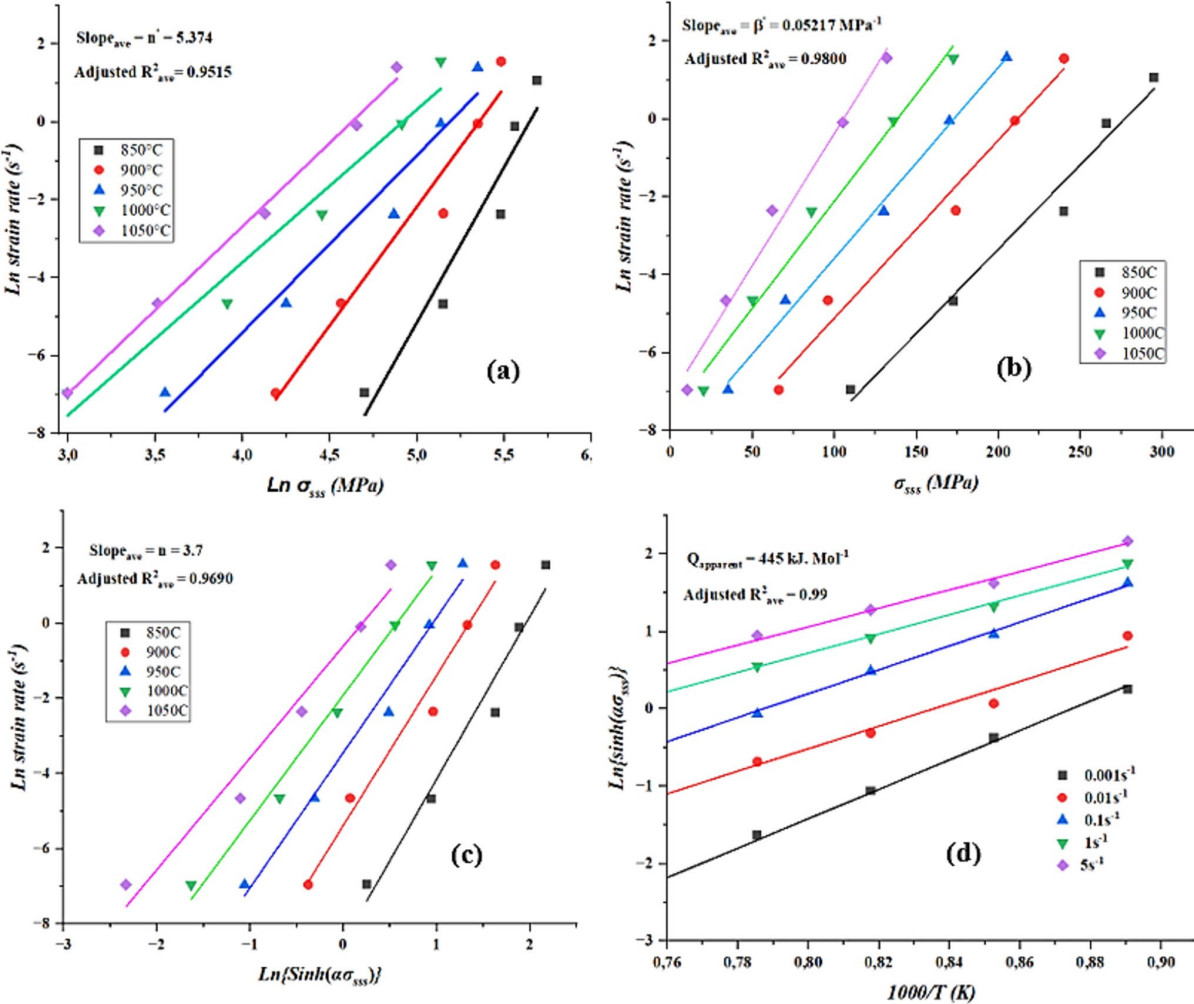
Linear plots used to calculate hot deformations constants at various hot working conditions.

Scatter plots of ln $\dot{\upvarepsilon }$ vs ln ${\sigma }_{sss}$ and ln $\dot{\upvarepsilon }$ vs ${\sigma }_{sss}$ using Eq. ((7) and Eq. ((8) respectively, followed by linear regression analysis yield linear curves given by Figure 7a and b below. By taking the average slope of each linear curve, the values of n′ = 5.374 and β = 0.05217 MPa^−1^ were obtained. Hence, the calculated α-value was averaged to 0.009708 MPa^−1^.

#### Computation of stress exponent (n)

The stress exponent is found by combining Eq. ((1) and Eq. ((2), then express natural logarithms on both sides of resulting expression to yield the following linear logarithmic equation:

9 $$\ln \dot{\varepsilon } = n\ln \left\{ {\sinh \left( {\alpha \sigma_{sss} } \right)} \right\} + \underbrace {{\ln A_{1} - \frac{Q}{RT}}}_{cons\tan t}$$

where $$n = \frac{{\partial \left( {\ln \dot{\varepsilon }} \right)}}{{\partial \ln \left\{ {\sinh \left( {\alpha \sigma_{sss} } \right)} \right\}}}\left| {_{T,\varepsilon } } \right. = gradientofthelinearcurveatcons\tan t{\text{Tan}} d\varepsilon$$

A scatter plot of ln $\dot{\upvarepsilon }$ vs $\text{ln}\left\{\text{sinh}\left(\alpha {\sigma }_{sss}\right)\right\}$ using Eq. ((9), followed by linear regression analysis gave linear curve of Figure 7c. Where the average slope of the linear curve was taken as the value of n = 3.7. The magnitude of n-value obtained seemed to suggest that the plastic deformation process was governed by gliding of dislocations^49^. Furthermore, since the strain rate sensitivity (m) is the reciprocal of n, a low value in the latter may mean better hot workability of 2205 DSS at high temperatures^15^.

#### Computation of activation energy (Q)

In finding the activation energy, Eq. ((9) is used, except that strain rate is maintained constant to yield the following expression:

10 $$\ln \left\{ {\sinh \left( {\alpha \sigma_{sss} } \right)} \right\} = \frac{Q}{Rn}\left( \frac{1}{T} \right) + \underbrace {{\frac{{\ln A - \ln \dot{\varepsilon }}}{n}}}_{cons\tan t}$$

where $$\frac{Q}{Rn} = \frac{{\partial \ln \left\{ {\sinh \left( {\alpha \sigma_{sss} } \right)} \right\}}}{{\partial \left( \frac{1}{T} \right)}}\left| {_{{\dot{\varepsilon },\varepsilon }} } \right. = gradientofthelinearcurveatcons\tan t\dot{\varepsilon }and\varepsilon$$

A scatter plots of ln{sinh∝(σ)} vs1000/T(K) and linear regression analysis were conducted at each strain rate to produce linear curves that are shown in Figure 7d. The slope of each linear curve was then multiplied by the universal gas constant (R) and stress exponent (n) to give activation energy (Q). By summing the activation energies from each linear curve and take the average, the activation energy for the plastic deformation process was found to be 445 kJ/mol. The calculated activation energy fell within those calculated by Farnoush et al.^17^, at low (310 kJ/mol) and high (554 kJ/mol) temperatures. In the same study^17^, it was also observed that the calculated activation energy was higher than those of single phase ferritic stainless steels but slightly less than austenitic ones.

#### Computation of dislocation structure (A_1_)

Once the plastic deformation activation energy is known, the Z- parameter values for each combination of T and $\dot{\varepsilon }$ were calculated using Eq. (1). Followed by introduction of naturals logs on both sides of Eq. (2) to get the following expression:

11 $$\mathit{ln}Z=\mathit{ln}{A}_{1}+nln\left\{\mathit{sin}h\left(\alpha \sigma \right)\right\}$$

where: ${A}_{1}$= intercept of the linear curve.

**Fig. 8:**
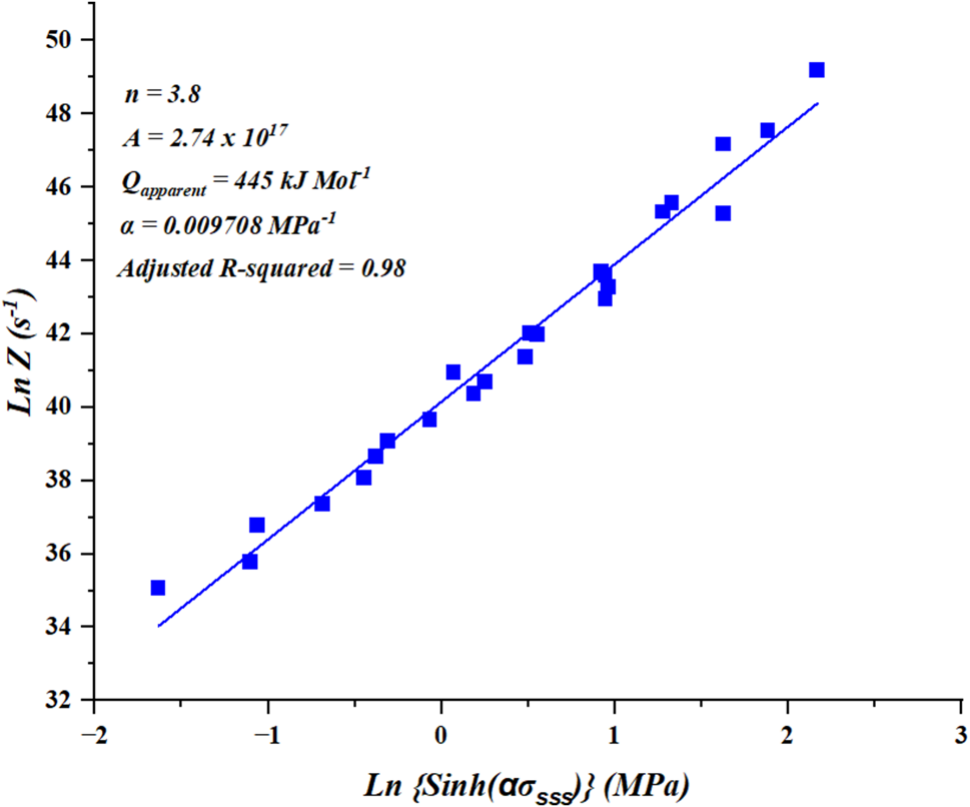
Linear plot for calculation of A1-constant.

A scatter plot of ln Z vs {sinh(∝σ)} and regression analysis at various hot working conditions yield a graph with single linear curve (Fig. 8). The linear intercept of the curve is equal to ${\text{lnA}}_{1}$ and performing the antilogarithm the constant A_1_ was found to be 2.74 × 10^17^ s^−1^.

A limited number of studies have been conducted on the hot deformation behaviour of 2205 DSS and the results of hot deformation constants obtained are given in Table 2. It can be noticed that the calculated value of α is approximately the same across all studies. The Q, ${A}_{1}$ and n were slightly different between the studies. The differences could be attributed to different hot testing conditions used during investigation or the choice of characteristics flow stress used when modelling^50,51^. According to McQueen et al.^52^, the only constant that tends to vary between the alloy of the same designation but taken from different melts is the ${A}_{1}$ because of variation in strength. However, the n and Q values should not vary much between studies of the alloy. This trend was observed in Table 2, where n and Q values did not vary much across the studies except the values of ${A}_{1}$.

**Table 2:** Hot deformation constants of 2205 DSS from various studies.

References	Hot deformation constants			
	α (MPa^−1^)	n	Q (kJ/mol)	${A}_{1}$(s^−1^)
*Evangelista* et al.^53^	*0.012*	*3.8*	*407*	*1.0* × *10*^*20*^
*Song* et al*.* ^54^	*0.00645*	*3.85*	*352*	*1.18* × *10*^*10*^
*Momeni and Dehghani* ^15^	*0.012*	*5.1*	*479*	*2.14* × *10*^*21*^
*Yang and Yan* ^55^	*0.012*	*6.62*	*460.9*	*1.51* × *10*^*20*^
*Farnoush *et al*.* ^17^	*0.012*	*4.2*	*432*	*–*
*Spigarelli *et al*.* ^56^	*0.012*	*4*	*474*	*–*
*Chen *et al*.* ^57^	*0.0083*	*4.48*	*405.8*	*2.02* × *10*^*16*^
*This research work*	*0.009708*	*3.7*	*445*	*2.74* × *10*^*17*^

#### Linear regression plots

##### Final steady state constitutive model

The calculated values of hot deformation constants were then inserted into Eq. (6) to yield a final constitutive model that predicts the steady state stress obtained from the experimental flow curves. The constants in Eq. ((12) were calculated using steady state obtained at various strain rates and temperatures. During steady state flow, the structural factor (A) remains unchanged and therefore the strain is not considered. Therefore, prediction of steady state stress or solving of Eq. ((*12*), at different strain rates and temperatures disregarded the true strain. In short, solving of Eq. ((12) was conducted by substituting Z-parameter calculated at different T (850 °C–1050 °C) and $\dot{\varepsilon }$ (0.001 s^-1^–5 s^−1^) into Eq. ((12).

12 $${\sigma }_{sss}= \frac{1}{0.009708}\mathit{ln}\left\{{\left(\frac{Z}{2.74X1{0}^{17}}\right)}^{\frac{1}{3.8}}+{\left[{\left(\frac{Z}{2.74X1{0}^{17}}\right)}^{\frac{2}{3.8}}+1\right]}^\frac{1}{2}\right\} \left(MPa\right)$$

Where$$Z = \dot{\varepsilon } \times \exp \left( {\frac{445000}{{8.3145T}}} \right)$$

**Fig. 9:**
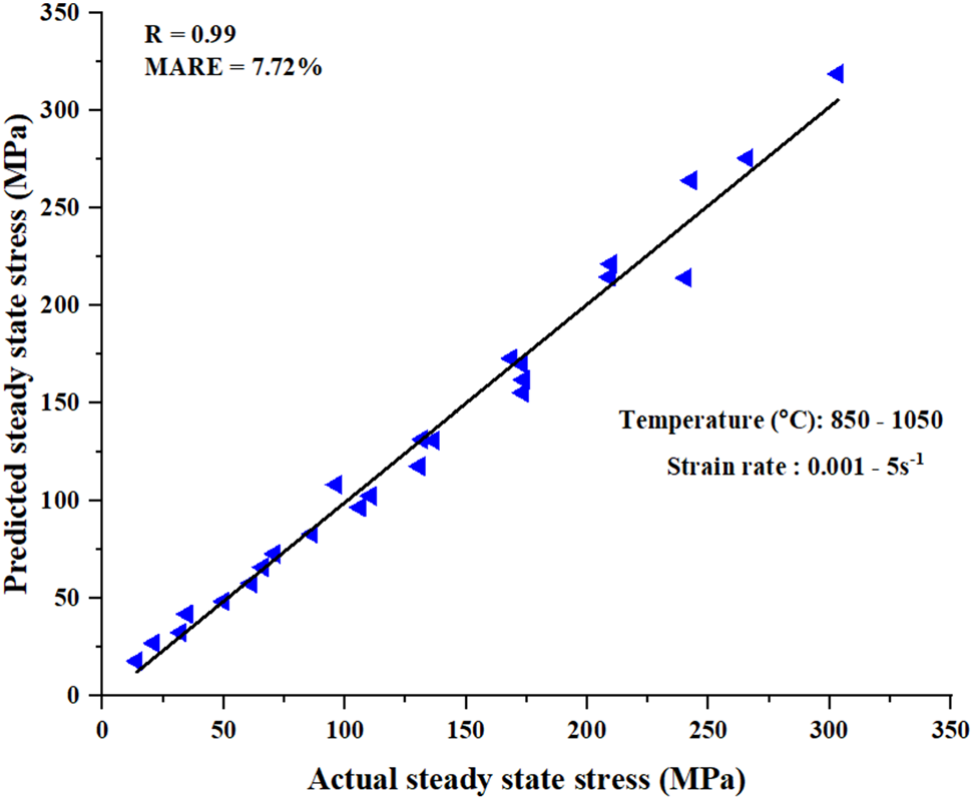
Actual vs predicted steady state stress.

The accuracy of the above model was verified by plotting the predicted steady state stress versus the experimental (actual) flow stress, then calculate the mean absolute relative error (MARE) using Eq. ((13) which was found to be 7.73%. The low value of MARE indicate that the model has a good predictability of steady state stress. A correlation coefficient (R) = 0.99 also indicated a strong positive linear relationship between the two stresses as shown in Figure 9.

13 $$Mare\left( \% \right) = \frac{1}{N}\sum\limits_{i = 1}^{N} {\left| {\frac{{M_{i} - C_{i} }}{{M_{i} }}} \right|} \times 100$$

where N = sample number, M_i_ = measured flow stress from the flow curves, C_i_ = calculated or predicted flow stress using Eq. ((12).

## Development of strain compensated Arrhenius rate type constitutive model

The constitutive model given in Eq. ((12) above is capable of predicting the characteristic flow stress and of which in this case it was steady state stress. In other words, the strain effect is not taken into account, which is valid because under steady state the flow stress is independent of true strain^58^. However, to be able to predict the flow curve from the work hardening region till steady state, Eq. ((12) has to be compensated with true strain^29,59^. The reason for this is that it can be well observed from the flow curves in Figure 4, that the flow stress does vary with true strain until the steady state region. The compensation is done by calculating the deformation constants (α, n, Q, and A_1_) from the presumable lowest true strain (e.g., ${\varepsilon }_{initial}$ = 0.05) to a total true strain (${\varepsilon }_{final})$ applied during hot compression tests. The computation of each constant using the procedure described in Section.  0, is usually done at every 0.05 true strain in the range of (ε = ${\varepsilon }_{initial}$, 0.1, 0.15…,${\varepsilon }_{final})$ in order to generate enough data for regression analysis. After the material constants are calculated as per above, they are scatter plotted as a function of true strain and fitted with high order polynomial. This yields empirical equations necessary to re-calculate the hot deformation constants at various true strains:

14 $$\left.\begin{array}{c}\begin{array}{c}\alpha \left(\varepsilon \right)={a}_{8}{\varepsilon }^{8}+{a}_{7}{\varepsilon }^{7}+{a}_{6}{\varepsilon }^{6}+{a}_{5}{\varepsilon }^{5}+{a}_{4}{\varepsilon }^{4}+{a}_{3}{\varepsilon }^{3}+{a}_{2}{\varepsilon }^{2}+{a}_{1}\varepsilon +{a}_{0}\\ n \left(\varepsilon \right)={b}_{8}{\varepsilon }^{8}+{b}_{7}{\varepsilon }^{7}+{b}_{6}{\varepsilon }^{6}+{b}_{5}{\varepsilon }^{5}+{b}_{4}{\varepsilon }^{4}+{b}_{3}{\varepsilon }^{3}+{b}_{2}{\varepsilon }^{2}+{b}_{1}\varepsilon +{b}_{0}\\ Q \left(\varepsilon \right)={c}_{8}{\varepsilon }^{8}+{c}_{7}{\varepsilon }^{7}+{c}_{6}{\varepsilon }^{6}+{c}_{5}{\varepsilon }^{5}+{c}_{4}{\varepsilon }^{4}+{c}_{3}{\varepsilon }^{3}+{c}_{2}{\varepsilon }^{2}+{c}_{1}\varepsilon +{c}_{0}\end{array}\\ \mathit{ln}{A}_{1}\left(\varepsilon \right)={d}_{8}{\varepsilon }^{8}+{d}_{7}{\varepsilon }^{7}+{d}_{6}{\varepsilon }^{6}+{d}_{5}{\varepsilon }^{5}+{d}_{4}{\varepsilon }^{4}+{d}_{3}{\varepsilon }^{3}+{d}_{2}{\varepsilon }^{2}+{d}_{1}\varepsilon +{d}_{0}\end{array}\right\}$$

where: ***a***, ***b***, ***c***, and ***d*** represent high order polynomial fitting coefficients.

High order polynomial Eqs ((14) serve the purpose of re-calculating material constants in the range of (ε = ${\varepsilon }_{initial}$, 0.1, 0.15…,${\varepsilon }_{final})$ at every 0.05 true strain interval. Eqs ((*14*) are then substituted into Eq. (6) to yield Arrhenius strain compensated model which is Eq. ((15) below:

15 $${{\varvec{\sigma}}}_{{\varvec{p}}{\varvec{r}}{\varvec{e}}{\varvec{d}}{\varvec{i}}{\varvec{c}}{\varvec{t}}{\varvec{e}}{\varvec{d}}}({\varvec{\varepsilon}})=\boldsymbol{ }\frac{1}{\boldsymbol{\alpha }({\varvec{\varepsilon}})}{\varvec{ln}}\left\{{\left(\frac{{\varvec{Z}}({\varvec{\varepsilon}})}{\boldsymbol{ }{\varvec{A}}({\varvec{\varepsilon}})}\right)}^{\frac{1}{{\varvec{n}}({\varvec{\varepsilon}})}}+{\left[{\left(\frac{{\varvec{Z}}}{\boldsymbol{ }{\varvec{A}}({\varvec{\varepsilon}})}\right)}^{\frac{2}{{\varvec{n}}({\varvec{\varepsilon}})}}+1\right]}^\frac{1}{2}\right\}\boldsymbol{ }({\varvec{M}}{\varvec{P}}{\varvec{a}})$$

where $$Z\left( \varepsilon \right) = \dot{\varepsilon } \times \exp \left( {\frac{Q\left( \varepsilon \right)}{{8.3145T}}} \right)$$

Eq ((15) allows calculation of the predicted flow stress at any true strain of interest.

### Determination of high order polynomial coefficients for hot deformation constants

**Fig. 10:**
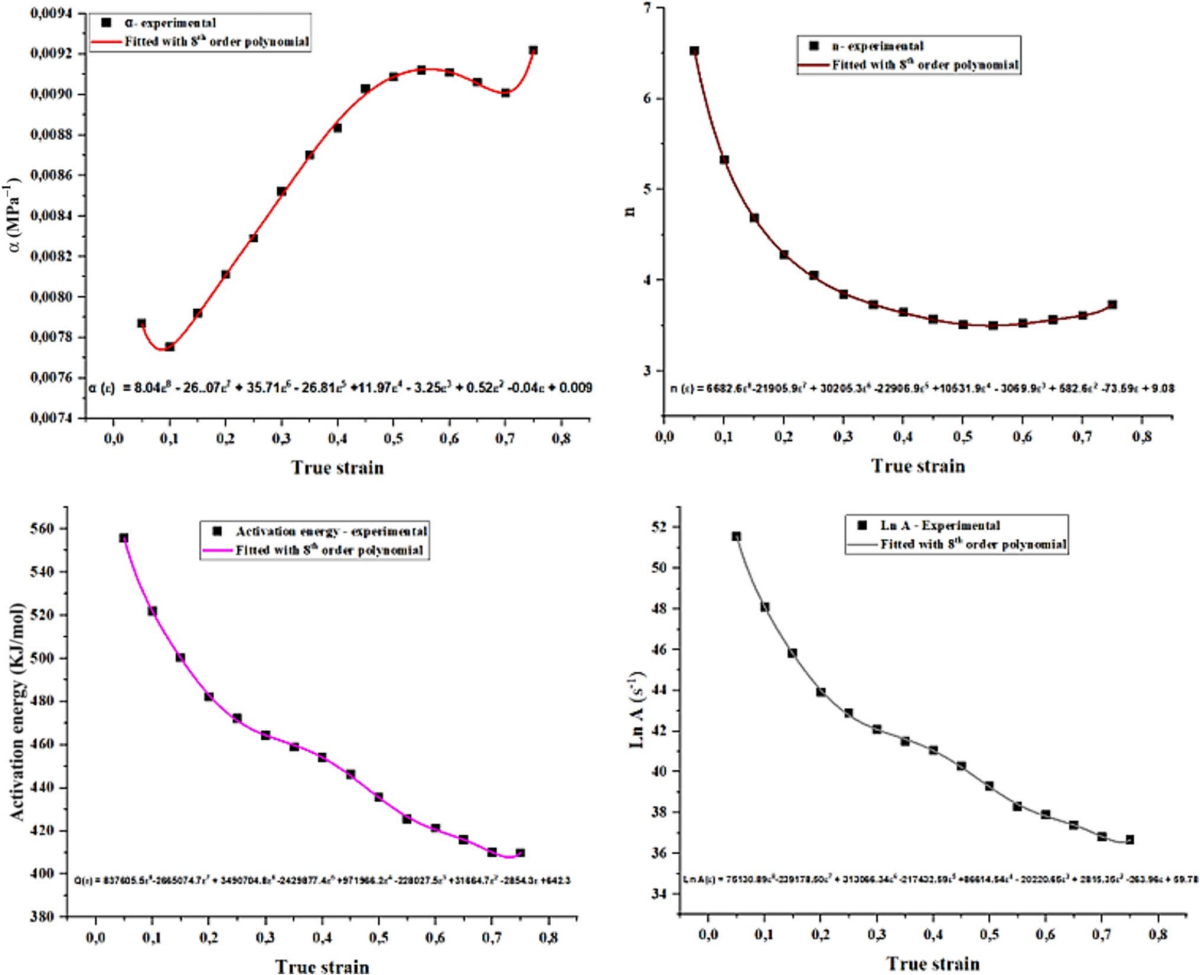
Polynomial curves of hot deformation constants as a function of true strain.

In this work, constitutive equations outlined above were used to compute the hot deformation constants at a true strain range of (0.05–0.75), strain rate (0.001–5 s^−1^) and temperature (850–1050 °C). After calculations, each deformation constant was scatter plotted as a function of true strain and then fitted with an 8th order polynomial to produce the polynomial curves given in Figure 10. High order polynomials bear no physical meaning rather than establishing an accurate mathematical relationship between hot deformation constants and plastic strain at various hot processing conditions. The fitting of 8th order was not unique to this study as Wang et al.^60^ also mentioned Zhao et al.^27^, Han et al.^61^, Xindi et.al.^62^ and Lei et al.^63^, who successfully use this order to establish α, n, LnA, Q = f(ε)). After fitting, the obtained polynomial curves in Figure 10 seemed comparable to other hot deformation studies of stainless steel conducted by Feng et.al.^26^ and Sen-Hao et.al.^26^.

### Interpretation of hot deformation constants

#### Stress multiplier (α)

Stress multiplier (α) increased till a true strain of approximately 0.75 and of which beyond this it reached a value of 0.009708 and became independent of applied true strain. According to Sheppard and Jackson^64^, the magnitude of α reflects the ability of the alloy to resist plastic deformation, whereby low values suggest difficulty in plastic deformation. In this work, α- increased within the true strain range of 0.18–0.55 indicating that the hot workability of the alloy was improving within this range.

#### Stress exponent (n)

The n-value is an inverse of strain rate sensitivity (m) that measures hot workability of the deforming alloy. For instance, low n-values implies high m and good hot workability. In support of stress multiplier results, the n-value also decreased within [0.18–0.55] true strain range before rising to a value of 3.8 at 0.8 true strain. Momeni et.al.^65^, also confirmed similar findings in a duplex stainless steel study where the observed minimum point in the fitted polynomial curve of n vs true strain (i.e. ε ≈ 0.55 in this study) marked the end of good hot workability. They^65^ explained that the cause of increase in n-value after reaching the minimum was due to shift in plastic deformation from soft ferrite into hard austenite phase. Thus, from the values of n and α, it can be inferred that the investigated alloy had a good hot workability within the true strain range of 0.18–0.55. These results also implied that deformation may have been accommodated by ferrite phase up to 0.55 true train, before shifting to harder austenitic phase^26,66^.

#### Activation energy (Q) and structural factor (A)

Activation energy (Q) also decreased with rise in true strain up 0.8, this was expected because strain accumulation raises store strain energy of the deforming alloy and subsequently the resistance or barrier to plastic deformation is lowered^66^. The strength of the alloy is embedded in constant A^52^, in this regard ${\text{lnA}}_{1}$ decreased with increase in true strain up to 0.8. This could be ascribed to additional heat brought by strain accumulation and leading to softening of the deforming alloy.

## Computation of predicted flow stress at various true strains

After fitting 8^th^ order polynomial as explained above, 8th order polynomial equations were derived for each deformation constants to determine coefficients of Eqs ((14) and the results are presented in Table 3.

**Table 3:** Polynomial coefficients for deformation constants.

α (MPa^−1^)	n	Q (kJ/mol)	Ln A (s^−1^)
${a}_{0}$=0.00898	${b}_{0}$=9.0759	${c}_{0}$=642.3240	${d}_{0}$=59.7755
${a}_{1}$=− 0.04165	${b}_{1}$=− 73.587	${c}_{1}$=2854.2880	${d}_{1}$=263.9606
${a}_{2}$=0.52209	${b}_{2}$=582.606	${c}_{2}$=31,664.730	${d}_{2}$=2815.382
${a}_{3}$=3.24731	${b}_{3}=3069.92$	${c}_{3}$=− 228,027.0	${d}_{3}$=20,220.60
${a}_{4}$=11.97341	${b}_{4}$=10,531.86	${c}_{4}$=971,966.20	${d}_{4}$=86,614.54
${a}_{5}$=− 26.8080	${b}_{5}$=22,906.90	${c}_{5}= 2429877$	${d}_{5}$=− 217,433
${a}_{6}$=35.71193	${b}_{6}$=3,205.34	${c}_{6}$=3,490,705.0	${d}_{6}$=313,066.3
${a}_{7}$=26.0715	${b}_{7}$=21,906.00	${c}_{7}$=2,665,075.0	${d}_{7}$=− 239,178
${a}_{8}$= 8.04215	${b}_{8}$= 6682.64	${c}_{8}$=837,605.50	${d}_{8}$=75,130.89

The obtained coefficients in Table.

Table 3 were used for calculation of deformation constants at a true strain range of interest [0.05 to 0.75], and Table .

Table 4 presents the results obtained.

**Table 4:** Hot deformation constants at various true strain strains for T: 850 °C–1050 °C and strain rate: 0.001 s^−1^–5 s^−1^.

True strain	α (ε)- MPa^−1^	n (ε)	Q (ε)-kJ/mol	Ln A(ε)-s^−1^
0.05	0.007864	6.53	555.64	51.57
0.10	0.007751	5.33	521.65	48.09
0.15	0.00791	4.69	500.01	45.80
0.20	0.008106	4.29	482.86	44.01
0.25	0.0083	4.03	471.02	42.78
0.30	0.008497	3.86	464.27	42.08
0.35	0.00869	3.74	459.74	41.61
0.40	0.008863	3.64	454.00	41.04
0.45	0.008996	3.57	445.49	40.22
0.50	0.009081	3.52	435.41	39.26
0.55	0.009117	3.50	426.54	38.41
0.60	0.009104	3.52	420.53	37.83
0.65	0.009047	3.57	415.91	37.39
0.75	0.009002	3.61	410.08	36.83
0.75	0.009209	3.73	409.90	36.66

The utilisation or solving of Eq. ((15) to predict flow stress at different strains, strain rate and temperature involved a series of steps including: (1) hot deformation constants displayed in Table .

Table 4 were calculated each strain by inserting coefficients from Table 3 into Eqs ((14), (2) The Z (ε) parameter incorporating different strain rate and temperature was also calculated at each level of strain, and (3) calculated hot deformation constants and the Z (ε) parameter were then substituted into Eq. ((15) to predict flow stress at each strain level. In summary, the predicted flow stress which in principle is solving of Eq. ((15) at different strain, strain rate and temperature was calculated by substituting Eqs ((14) and Eq. ((*1*) into Eq. ((15). As an example, Eq. ((*16*) shows how Eqs ((14) and Eq. ((1) were inserted into Eq. ((15) to predict the flow stress at 0.05 true strain. This process was repeated until 0.75 true strain and Table Table 5 shows the predicted stress values at a temperature and strain rate of 850 °C and 0.01 s^−1^ respectively. Similar tables to Table Table 5 were generated at different strain rates (0.001–5 s^−1^) and temperatures (850 °C–1050 °C). The results obtained were then used to generate predicted flow curves at different hot processing conditions.

**Table 5:** Predicted flow stress in (MPa) at various true strains and a temperature of 850 °C and strain rate of 0.01 s^−1^.

True strain	α (MPa^−1^)	n	Q (kJ/mol)	Ln A (s^−1^)	Z	Predicted stress (MPa)
0.05	0.007864	6.53	555.64	51.57	7.01E + 23	163.34
0.1	0.007751	5.33	521.65	48.09	1.84E + 22	175.20
0.15	0.00791	4.69	500.01	45.80	1.81E + 21	180.18
0.2	0.008106	4.29	482.86	44.01	2.89E + 20	181.42
0.25	0.0083	4.03	471.02	42.78	8.12E + 19	181.10
0.3	0.008497	3.86	464.27	42.08	3.94E + 19	180.08
0.35	0.00869	3.74	459.74	41.61	2.43E + 19	178.34
0.4	0.008863	3.64	454.00	41.04	1.31E + 19	175.66
0.45	0.008996	3.57	445.49	40.22	5.27E + 18	172.14
0.5	0.009081	3.52	435.41	39.26	1.79E + 18	168.38
0.55	0.009117	3.50	426.54	38.41	6.93E + 17	165.23
0.6	0.009104	3.52	420.53	37.83	3.64E + 17	163.16
0.65	0.009047	3.57	415.91	37.39	2.22E + 17	161.79
0.7	0.009002	3.61	410.08	36.83	1.19E + 17	160.04
0.75	0.009209	3.73	409.90	36.66	1.17E + 17	157.99

16 $${{\varvec{\sigma}}}_{{\varvec{p}}{\varvec{r}}{\varvec{e}}{\varvec{d}}{\varvec{i}}{\varvec{c}}{\varvec{t}}{\varvec{e}}{\varvec{d}}}=\frac{1}{0.007864}{\varvec{ln}}\left\{{\left(\frac{\dot{{\varvec{\varepsilon}}}\boldsymbol{*}{\varvec{e}}{\varvec{x}}{\varvec{p}}\left(\frac{555640}{8.314{\varvec{T}}}\right)}{1.131{{\varvec{e}}}^{18}}\right)}^{\frac{1}{6.53}}+{\left[{\left(\frac{\dot{{\varvec{\varepsilon}}}\boldsymbol{*}{\varvec{e}}{\varvec{x}}{\varvec{p}}\left(\frac{555640}{8.314{\varvec{T}}}\right)}{{1.131{\varvec{e}}}^{18}}\right)}^{\frac{2}{6.53}}+1\right]}^\frac{1}{2}\right\}\boldsymbol{ }({\varvec{M}}{\varvec{P}}{\varvec{a}})$$

### Experimental vs predicted flow stress curves

**Fig. 11:**
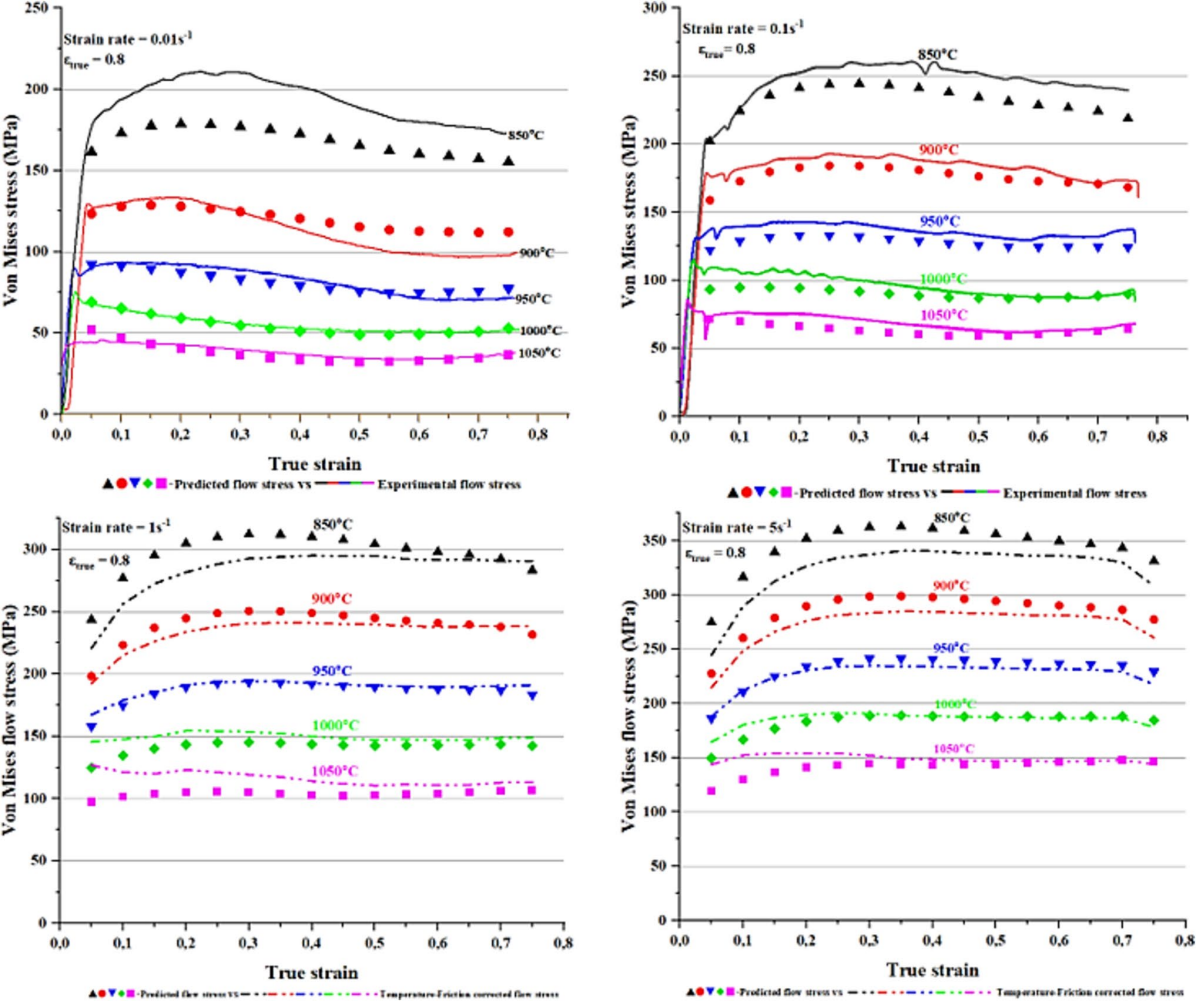
Experimental flow curves vs predicted flow curves.

The predicted flow stress values calculated at different strain, strain rate and temperature using Eq. ((15) were plotted as a function of true strain to produce modelled flow curves. These curves were then superimposed to the experimental ones for comparison as shown in Figure 11. The predictive model seemed to show a reasonable accuracy between 900 and 1050 °C across all the strain rates.

A noticeable discrepancy between predicted and experimental flow stress was observed at a temperature of 850 °C across all the strain rates. For instance, at a lower strain rate (0.01 and 0.1 s^−1^) the model underestimated the values of predicted flow stress, whereas the opposite was seen at high strain rates (1 and 5 s^−1^) and the same temperature. A cause of deviation could be linked to slightly variation in the value of α, which brought a significant change in predicted values^67^. This implied that, small changes in material’s constants (β and n’) brought a change in the value of α and subsequently a big deviation in predicted flow stress from the actual was observed. In addition, a deformation carried out at higher strain rates or low temperatures may introduce deformation heating, which brings additional softening of the alloy and as a result the actual flow stress values appear lower than normal.

The accuracy of the empirical models in predicting the hot flow curves of alloys is limited, this is because the dislocation dynamics governing the plastic deformation process are disregarded^68^. Despite the limited accuracy, these models have been applied in several studies^10,26,28,29,59,69^ to model the hot flow curves. According to Momeni and Dehghani^65^ the preference of phenomenological (empirical) models over thermodynamic and kinetics based ones (physical-based models) is due to their ability to predict the entire flow curve. Whereas, physical based models like those of Enstrin-Mecking^70^ only predict the segment part of the flow curve which is either the work hardening + DRV or DRX region. Zhao et al.^27^, also supported the preference the empirical models because of their simplicity in terms of material constants calculations. They^27^, argue that physical based models require too much computation of thermodynamic and kinetic parameters and thus they can be difficult to apply. In another study by Kingklang and Uthaisangsuk^17^, physical models were deemed as costly and complex whereas empirical models were simple and reliable for engineering applications. The other non-linear estimation model not requiring complex thermodynamics and kinetics properties and yet precise for estimation of flow curves was that of Shafiei et al.^71^. The advantage of their model^71^ was that it predicts the flow stress in the work-hardening (WH) + DRV and DRX regions using flow characteristics flow stress points that could easily be derived from the flow curves. In another study, Shafiei and Dehghani^72^ developed a new constitutive model for prediction of hot flow curves using logarithmic power function. Their^72^ findings showed that the proposed model gave a better estimation of flow curves when compared with other previous methods.

### Predictive strength of the model

**Fig. 12:**
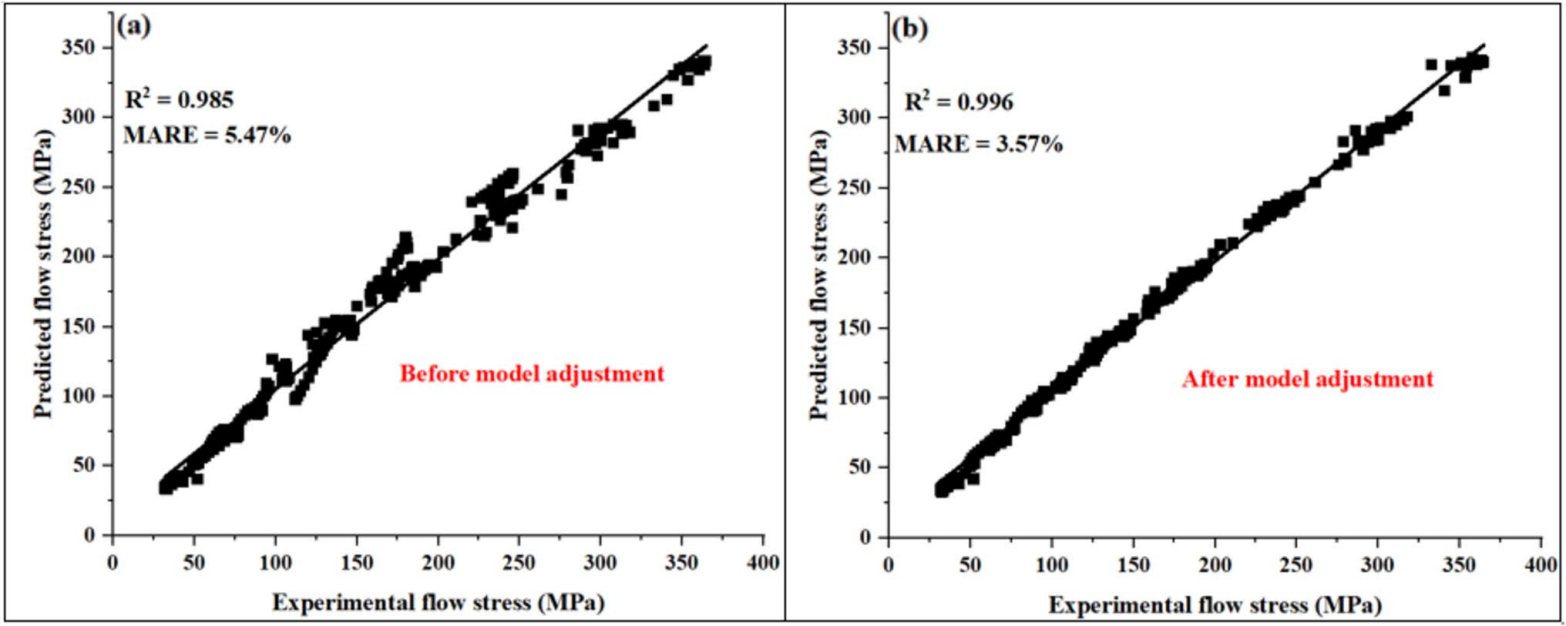
Experimental vs predicted flow stress at ε [0.05–0.75], T [850–1050 °C], and ε ˙ [0,01, 0,1, 1, 5] s-1 (**a**) before model adjustment, and (**b**) after model adjustment.

The model’s accuracy was assessed by first scatter plotting experimental vs predicted flow stress within the range of [0.05–0.75] true strain as illustrated in Figure 12a below. This was followed by performing a linear regression analysis through fitted data to obtain the R^2^ value which was found to be 0.984. Based on this R^2^ value, there was a good linear relationship between fitted data. Furthermore, the overall percentage error between experimental and predicted flow stress was assessed through calculation of mean average relative error (MARE) using Eq. ((*13*). The MARE was found to be 5.47% and of which this suggested that the predictive model had a good reasonable accuracy.

## Refinement of the predictive model

**Fig. 13:**
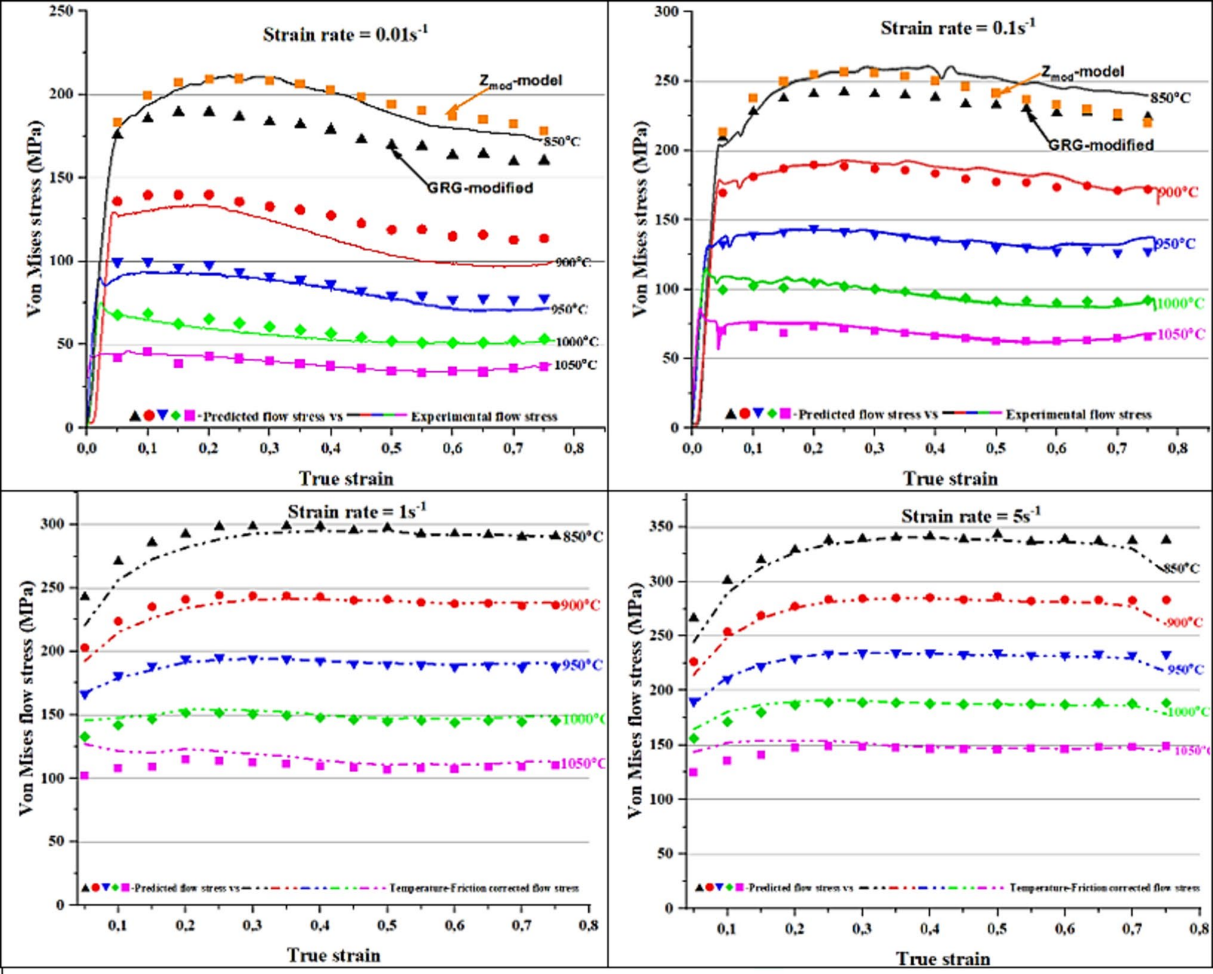
Experimental flow curves vs predicted flow curves after GRG optimisation.

Despite the high value of R^2^ and low value of MARE as indicated by Figure 12a, noticeable deviations between predicted and measured flow stress were well observed in Figure 11. Notably, the discrepancy was more severe between 850 °C and 900 °C across all the strain rates and of which this necessitated the refinement of the model to improve its accuracy. In this work, refinement was done using non-linear generalised reduced gradient (GRG) method which is an optimising tool that is embedded in solver Microsoft excel. GRG was quite easy to apply as it required few inputs to perform optimisation at a relatively short time. However, certain parameters had to be defined before optimisation including identification of objective function, variables to be optimized and constraints. Since the aim was to minimise or eliminate discrepancies between predicted and experimental flow stress, it was logical to assign MARE as an objective function, hot deformation constants (α, n Q and A) as variables to be optimised and R the universal gas constant as a constraint. The optimisation of variables was performed at each true strain and range of temperature [850–1050 °C] and strain rates [0.01, 0.1, 1 and 5] s^-1^. During optimisation it was noticed that within the steady state regions, the variables being optimised did not show significant change from the original values. This could be attributed to the independence of flow stress with strain at the steady state region. Table 6 shows the deformation constants obtained at each strain after performing GRG optimisation. From these optimised deformation constants, re-calculation of predicted flow stress was conducted at each true strain and estimated flow curves replotted as shown in Fig. Figure 13. In this figure, it can be seen that the GRG refinement did improve the accuracy of the model as most flow curves showed minimal discrepancy. MARE also decreased from 5.47% to 3.57% meaning that the accuracy of model improved by 34.7% (see Figure 12b). In addition, improvement in linear relationship between the fitted data was noticed through increase in R^2^ value from 98.56% to 99.6%.

**Table 6:** Hot deformation constants after GRG optimisation.

True strain	α (MPa^−1^)	n	Q (kJ/mol)	Ln A (s^−1^)
0.05	0.021758	3.14	597.91	49.60
0.1	0.012894	4.14	553.81	47.72
0.15	0.014045	3.39	529.78	45.49
0.2	0.011882	3.73	502.75	43.47
0.25	0.010676	3.80	484.13	42.39
0.3	0.010571	3.73	474.91	41.68
0.35	0.01064	3.65	471.24	41.41
0.4	0.0104	3.63	464.86	41.03
0.45	0.01047	3.53	449.26	39.66
0.5	0.009981	3.52	440.52	39.18
0.55	0.011148	3.29	436.67	38.33
0.6	0.009881	3.51	424.74	37.78
0.65	0.010461	3.37	421.60	37.20
0.7	0.009312	3.64	410.26	36.64
0.75	0.009193	3.69	410.36	36.66

Regardless of GRG refinement, the flow curves belonging to strain rates of 0.01 and 0.1 s^–1^ at temperature of 850 °C showed slightly improvement. This observation seemed to indicate that there may be some new deformation mechanisms controlling the flow stress as the temperature descend to 850 °C and of which the current model may have not been capable to capture since it was empirical based. In light of this persisting discrepancy at mentioned deformation conditions, Eq. ((1) was multiplied on both sides by ${\dot{\varepsilon }}^{-5/4}$ to compensate for strain rate and modified Z-parameter. This approach was also applied Peng et.al, ^73^ and Lin et al.^74^, in an effort to improve the accuracy of strain compensated Arrhenius rate type model. The -5/4 exponent was the most suitable for modification of Z parameter after trying other different values (i.e., 1/3, -1/3, 1/4, 1/2). Therefore, after compensating for strain rate the modified Z-parameter (${\text{Z}}_{\text{mod}}$) worked out as follows:

17 $${Z}_{mod}={\dot{\varepsilon }}^{\frac{-1}{4}}\mathit{exp}\left(\frac{Q}{RT}\right)$$

Eq. ((17) was then incorporated into Eq. ((15) to yield Eq. ((18) below for prediction of new flow stresses. The flow curves in Figure 13 corresponding to 850 °C and strain rates of 0.01 and 0.1 s^-1^ were re-produced using the original deformation constants in Table .

Table 4 and Eq. ((18). As can be seen, a significant improvement was realised at these deformation conditions after the modification of Z-parameter. The negative relation between Z-parameter and strain rate at low temperature still require further investigation as mentioned previous studies^73,75^ compensated strain rate with positive exponent.

18 $${\sigma }_{predicted}(\varepsilon )= \frac{1}{\alpha (\varepsilon )}\mathit{ln}\left\{{\left(\frac{{Z}_{mod}(\varepsilon )}{ A(\varepsilon )}\right)}^{\frac{1}{n(\varepsilon )}}+{\left[{\left(\frac{{Z}_{mod}(\varepsilon )}{A (\varepsilon )}\right)}^{\frac{2}{n(\varepsilon )}}+1\right]}^\frac{1}{2}\right\} (MPa)$$

## Comparison of the current model with non-linear estimation model

As alluded before that Shafiei et al.^71^, proposed a non-linear estimation model for prediction of flow curves during hot working. From their work^71^, they proposed that the flow stress due to WH + DRV and DRX could be estimated using the following models:

### WH + DRV

19 $${\sigma }_{(DRV)}={\sigma }_{c}+{\theta }_{c}\left[\frac{{\varepsilon }_{c}{\varepsilon }_{s}}{{\varepsilon }_{s}-{\varepsilon }_{c}}\left(1+\mathit{ln}\left(\frac{\varepsilon }{{\varepsilon }_{c}}\right)-\left(\frac{\varepsilon }{{\varepsilon }_{c}}\right)\right) +(\varepsilon - {\varepsilon }_{c})\right]$$

### DRX

20 $$\sigma_{{\left( {DRX} \right)}} = \sigma_{{\left( {DRV} \right)}} - \left( {\sigma_{s} - \sigma_{ss} } \right) \times X_{DRX}$$

where: ${\sigma }_{\text{c}}$= critical stress, ${\sigma }_{\text{s}}$ = saturated stress, ${\sigma }_{\text{ss}}$ = stead-state stress, ${\uptheta }_{\text{c}}$= critical work-hardening rate, ${\upvarepsilon }_{\text{c}}$ = critical strain, and ${\varepsilon }_{\text{s}}$ = saturated strain.

**Fig. 14:**
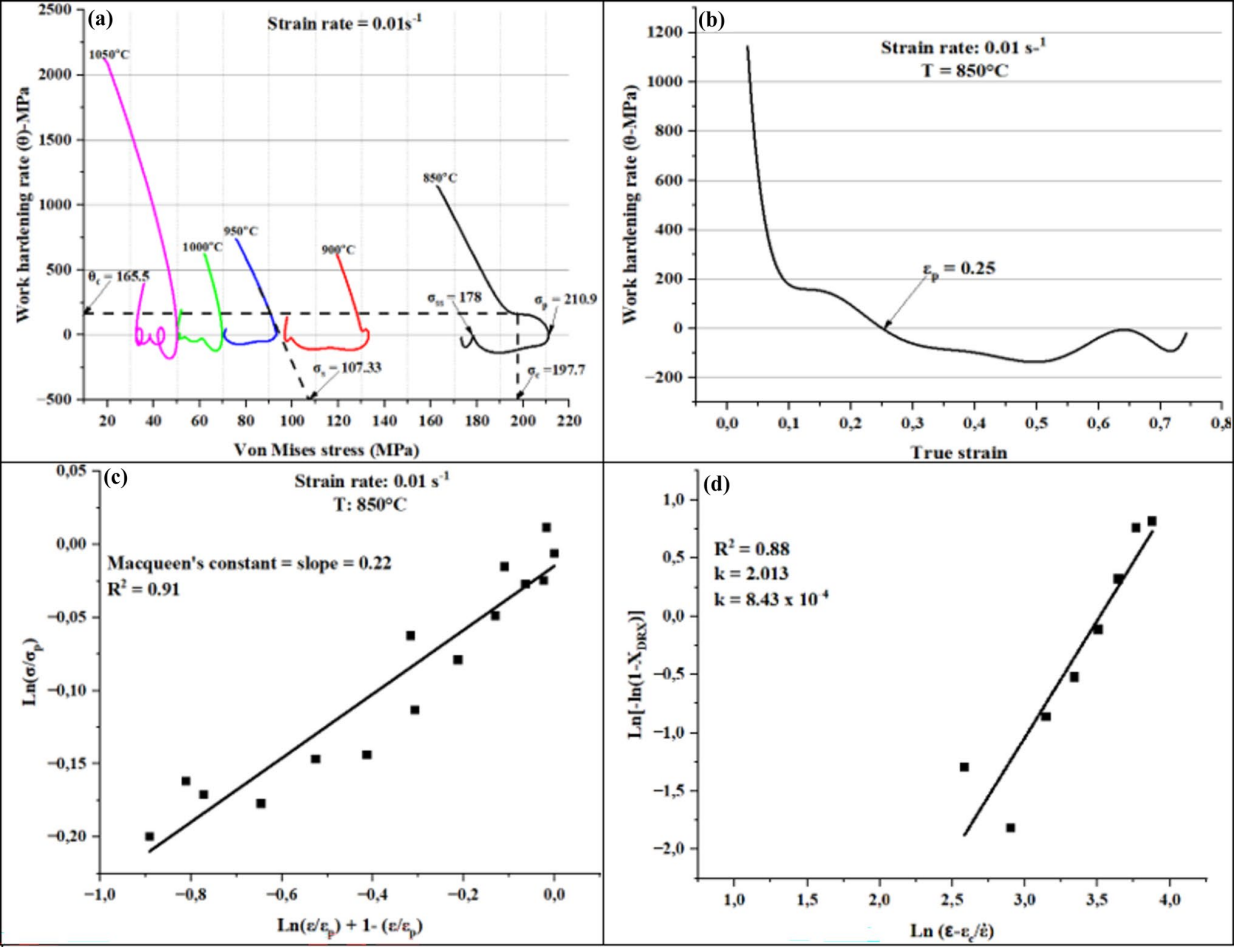
(**a**) WHR vs flow stress, (**b**) WHR vs true strain, (**c**) Macqueen’s plot for determination of C-constant, and (**d**) JMAK) plot for determination of j and k.

In this work, a comparison was made between non-linear estimation model proposed by Shafiei et al.^71^, and strain compensated Arrhenius typed model using flow curves generated at a strain rates of 0.01 s^–1^ and 5 s^–1^. Prior estimation of flow curves, it was necessary to determine all the parameters found in Eq. ((19) and Eq. ((20). To illustrate how the parameters were obtained a strain rate of 0.01 s^–1^ was used as an example, then the same procedure adapted for other investigated strain rates. Firstly, the peak stresses (σ_p_) at each deformation condition were determined using a plot of work hardening rate (WHR) vs flow stress as shown in Figure 14a^76,77^. After finding the peak stresses, the corresponding peak strains (ε_p_) were also determined from plot of WHR vs true strains (see Figure 14b. In order to find the critical strain, the Cingara equation given below in Eq. ((21) for estimation of flow stress up to the peak point was applied^78^. From this equation, the Macqueen’s constant (C) was estimated by plotting Ln (σ/σ_p_) vs Ln (ε/ε_p_) + 1- (ε/ε_p_) then perform a linear fit to find a slope which was equal C (see Figure 14c).

21 $$\sigma = \sigma_{p} [(\varepsilon /\varepsilon_{p} )\exp (1 - (\varepsilon /\varepsilon_{p} ))]^{C}$$

After computation of C, the normalised critical strain was determine using Eq. ((*22*):

22 $$\frac{{\varepsilon }_{c}}{{\varepsilon }_{p}}=\frac{\sqrt{1-C} -(1-C)}{C}$$

Following the computation of critical strains, the corresponding critical stresses were obtained from the flow curves and thereafter the critical work hardening from WHR vs flow stress was determined (see Figure 14a). The X_DRX_ which is the amount of fractional softening after the peak stress due to DRX was calculated from the flow curves using Eq. ((*23*) below^79,80^:

23 $${X}_{(DRX)}= \frac{{\sigma }_{s} - \sigma }{{\sigma }_{s} - {\sigma }_{ss}}$$

where σ_s_-σ = measure the drop in flow stress from the saturated stress to any flow stress point in a flow curve after the peak stress and σ_s_-σ_ss_ = expected maximum drop in flow stress.

X_DRX_ can also be calculated using a well know Avrami model for kinetics that is given by following Eq. ((*24*) below^77,81,82^:

24 $${X}_{(DRX)}= 1- exp \left(-j{\left(\frac{\varepsilon - {\varepsilon }_{c}}{\dot{\varepsilon }}\right)}^{k}\right)$$

where: j = material constant that is dependent on the chemical composition, and k = Avrami constant that measures the kinetics of DRX and dependent on the hot processing parameters.

The values of j and k can be determined by first introducing natural logarithms on both sides of Eq. ((24) to yield Eq. ((25) below.

25 $$\mathit{ln}\left[-\mathit{ln}(1-{X}_{DRX})\right]=\mathit{ln}j+k\mathit{ln}\left(\frac{\varepsilon - {\varepsilon }_{c}}{\dot{\varepsilon }}\right)$$

Then a scatter plot of $\text{ln}\left[-\text{ln}(1-{\text{X}}_{\text{DRX}})\right]$ vs $\text{ln}\left(\frac{\upvarepsilon - {\varepsilon }_{c}}{\dot{\varepsilon }}\right)$, followed by a linear fit to get a slope and intercept that represents k and ln j respectively^79^. Fig. 14d shows Johnson–Mehl–Avrami-Kolmogorov (JMAK) plot with the values of j and k which were subsequently inserted into Eq. ((24) for calculation of X_DRX_ at various strains.

**Fig. 15:**
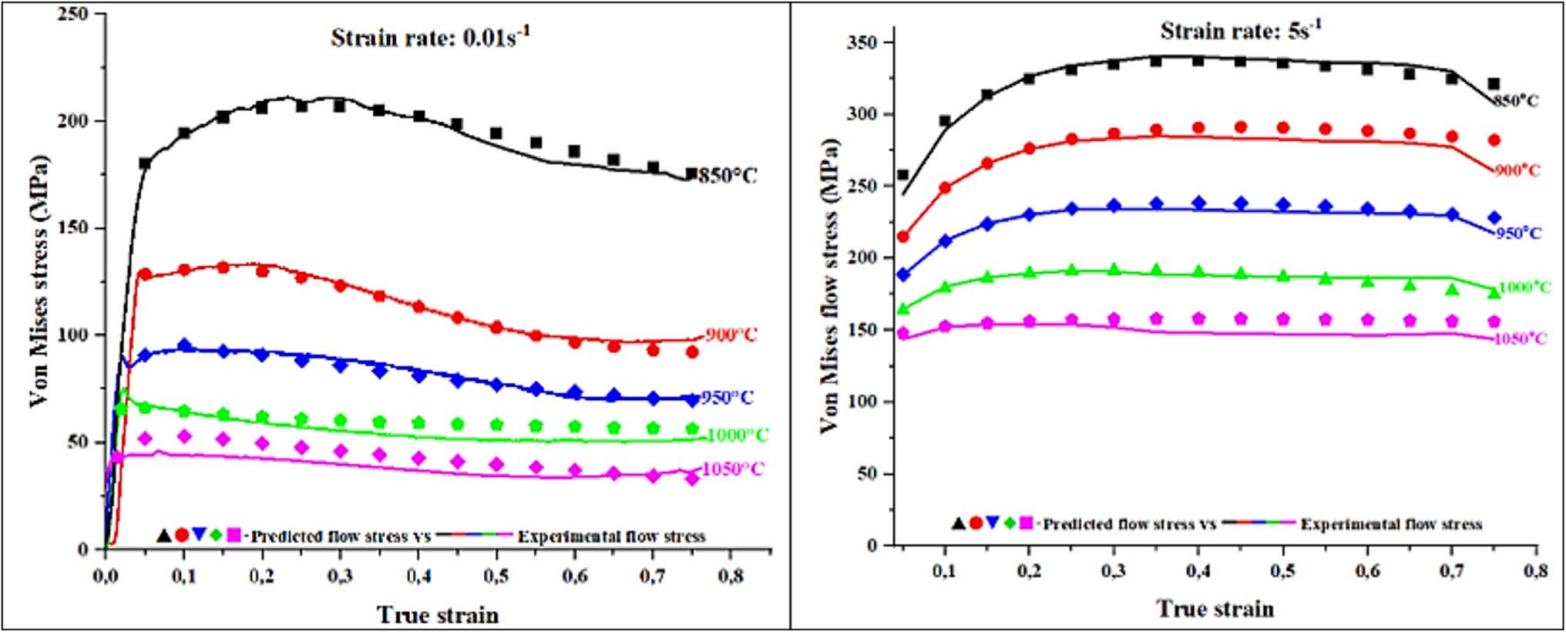
Flow curves of 2205 DSS predicted suing non-linear estimation model.

The above whole procedure was performed at each deformation condition to determine the relevant parameters for Eq. ((19) and Eq. ((20) and the obtained results are presented in Table Table 7. The calculated parameters were then inserted into Eq. ((19) and Eq. ((20) for calculation of flow stress at various true strains, and the resulting predicted flow curves are shown in Figure 15. It can be observed from this figure that non-linear models are more robust than strain-compensated Arrhenius rate type model in terms of accuracy when used for estimation of flow curves. The reason for such precision in non-linear models could be attributed to the fact that they employed flow stress characteristics points from the flow curves which are govern by active deformation mechanisms at the particular deformation condition. Whereas strain-compensated Arrhenius rate type model is solely empirical and tend to exclude mechanisms or parameters that govern the response of the deforming alloy during hot working. To extend the applicability of non-linear models over a wide range of deformation conditions, most of the parameters in Eq. ((*19*) and Eq. ((*20*) are temperature and strain rate dependent, as a result they should be expressed as a function of Z-parameter.

**Table 7:** Experimental data used in prediction of 2205 DSS flow curves.

Alloy	$\dot{\varepsilon }$(s^−1)^	T (°C)	C	${\varepsilon }_{C}$	${\sigma }_{C}$(MPa)	${\theta }_{C}$(MPa)	${\sigma }_{s}$(MPa)
2205 DSS	0.01	850	0.22	0.117	197.7	165.50	220
	0.01	900	0.14	0.089	130.39	31.31	139
	0.01	950	0.10	0.046	90.20	158.75	105.76
	0.01	1000	0.03	0.019	65.95	371.53	76.79
	0.01	1050	0.02	0.015	43.14	738	60
	5	850	0.28	0.180	320.98	200.82	375
	5	900	0.25	0.172	271.33	208.68	311
	5	950	0.20	0.147	223.05	179.60	263.91
	5	1000	0.04	0.125	183.04	135.82	220
	5	1050	0.04	0.101	152.69	60.78	180

## Conclusions

- The temperature and strain rate largely influence the response of 2205 DSS during hot deformation. For instance, flow curves showed that low temperature and high strain rate increased the flow stress and vice versa.
- The calculated hot deformation constants during steady state (α = 0.009708, Q = 445 kJ/mol, n = 3.7 and A = 2.74 × 10^17^) did not differ significantly from the previous studies of the same alloy. The alloy showed a good hot workability between 0.18—0.55 true strain range as reflected by decrease n and increase in α -value within this range.
- The polynomial curves show that true strain has a greater influence on the applied stress as indicated by change in material’s constant with variation in strain.
- The steady state predictive model showed a good estimation of steady stress through a low value of MARE = 7.73% and excellent linear relationship between the fitted data as indicated by high R^2^ value that was ≈ 0.99.
- The original strain-compensated Arrhenius rate-type constitutive model gave a good estimation of flow curves from 900 to 1050 °C across all the strain rates. However, at 850 °C and for all strain rates there was a discrepancy between predicted and experimental flow stress. And despite this discrepancy low value of MARE ≈ 5.47% and high R^2^ value ≈ 0.984 showed that the model was well capable of estimating flow curves. Generalised reduced gradient (GRD) method improved the accuracy of the model by 34.7% but noticeable deviation still persisted at 850 °C and strain rate of 0.01 s^–1^ and 0.1 s^–1^.
- Modification of Z-parameter by compensating the strain rate with a certain exponent improved the accuracy of the model at low temperatures.
- Non-linear models not requiring calculations of thermodynamic and kinetics properties appeared more robust than empirical models simply because they relied on characteristics flow stress points governed by underlying deformation mechanisms.

## Data Availability

The datasets used and/or analysed during the current study available from the corresponding author on reasonable request.
